# BRG1 promotes progression of B-cell acute lymphoblastic leukemia by disrupting PPP2R1A transcription

**DOI:** 10.1038/s41419-024-06996-w

**Published:** 2024-08-26

**Authors:** Qian Kang, Dan Ma, Peng Zhao, Xiao Chai, Yi Huang, Rui Gao, Tianzhuo Zhang, Ping Liu, Bo Deng, Cheng Feng, Yan Zhang, Yinghao Lu, Yanju Li, Qin Fang, Jishi Wang

**Affiliations:** 1 Medical College, Soochow University, Suzhou, 215006 China; 2 Department of Hematology, The Affiliated Hospital of Guizhou Medical University, Guiyang, 550004 China; 3 National Clinical Research Center for Hematologic Diseases, the First Affiliated Hospital of Soochow University, Suzhou, 215006 China; 4 Hematopoietic Stem Cell Transplantation Center of Guizhou Province, Key Laboratory of Hematological Disease Diagnostic & Treat Centre of Guizhou Province, The Affiliated Hospital of Guizhou Medical University, Guiyang, 550004 China; 5 Department of Pharmacy, Affiliated Hospital of Guizhou Medical University, Guiyang, 550004 China

**Keywords:** Oncogenesis, Transcription, Acute lymphocytic leukaemia

## Abstract

Despite advancements in chemotherapy and the availability of novel therapies, the outcome of adult patients with B-cell acute lymphoblastic leukemia (B-ALL) remains unsatisfactory. Therefore, it is necessary to understand the molecular mechanisms underlying the progression of B-ALL. Brahma-related gene 1 (BRG1) is a poor prognostic factor for multiple cancers. Here, the expression of BRG1 was found to be higher in patients with B-ALL, irrespective of the molecular subtype, than in healthy individuals, and its overexpression was associated with a poor prognosis. Upregulation of BRG1 accelerated cell cycle progression into the S phase, resulting in increased cell proliferation, whereas its downregulation facilitated the apoptosis of B-ALL cells. Mechanistically, BRG1 occupies the transcriptional activation site of PPP2R1A, thereby inhibiting its expression and activating the PI3K/AKT signaling pathway to regulate the proto-oncogenes c-Myc and BCL-2. Consistently, silencing of BRG1 and administration of PFI-3 (a specific inhibitor targeting BRG1) significantly inhibited the progression of leukemia and effectively prolonged survival in cell-derived xenograft mouse models of B-ALL. Altogether, this study demonstrates that BRG1-induced overactivation of the PPP2R1A/PI3K/AKT signaling pathway plays an important role in promoting the progression of B-ALL. Therefore, targeting BRG1 represents a promising strategy for the treatment of B-ALL in adults.

## Introduction

B-cell acute lymphoblastic leukemia (B-ALL) is a haematologic malignancy in which lymphocytes exhibit incomplete differentiation and spread to the bone marrow, blood and extramedullary sites. Approximately 75% of acute lymphoblastic leukemia (ALL) cases are attributed to B-ALL. With limited treatment options and a cure rate of < 40%, adult B-ALL has historically had a poorer prognosis than paediatric ALL, which is curable in > 90% of cases [1, 2]. Notably, among patients aged > 50 years, only 25% of patients are alive 5 years after diagnosis, suggesting that further improvements are warranted to identify novel oncogenes and optimise therapies for older patients with B-ALL ( ≥ 40 years of age) [3, 4].

With recent advancements in high-throughput gene chip and next-generation sequencing technology, numerous novel genetic alterations have been identified [5–7]. Remarkably, epigenetic alterations have been identified as novel and crucial factors involved in the development of ALL. Unlike gene mutations, epigenetic modifications are reversible. Therefore, they serve as a promising therapeutic target for leukemia [8, 9].

ATP-dependent chromatin remodeling, regulated by chromatin remodeling complexes, is one of the epigenetic mechanisms that regulate gene expression during mammalian development. Brahma-related gene 1 (BRG1) is encoded by the SWI/SNF-related matrix-associated actin-dependent regulator of chromatin, subfamily A, member 4 (SMARCA4) gene, which is one of the two mutually exclusive catalytic ATPase subunits in the switch/sucrose-nonfermentable (SWI/SNF) complex. It performs hydrolysis to supply energy to the SWI/SNF chromatin-remodeling complex, which functions synergistically with transcription factors to mobilise nucleosomes to activate or inactivate specific genes [10–12]. Several studies have reported that BRG1 is one of the most frequently mutated chromatin remodeling ATPase in cancer and may be involved in disease progression as an oncogene [13–15]. In colorectal cancer (CRC) [16], hepatocellular carcinoma (HCC) [17] and acute myeloid leukemia (AML) [18], BRG1 overexpression facilitates the driving of oncogenic transcriptional processes that affect the proliferative capability of cancer cells. Recently, we found that BRG1 expression was markedly higher in patients with first-diagnosed B-ALL than in healthy individuals in the Gene Expression Omnibus (GEO) and The Cancer Genome Atlas (TCGA) datasets. In addition, BRG1 knockdown inhibited the proliferation of the REH cells as described by Shi et al. [19]. Based on the findings of these two studies, we speculated that BRG1 might be involved in the pathogenesis of B-ALL. However, the precise role of BRG1 and the mechanisms underlying its overexpression in B-ALL remain elusive.

This study reveals the role of BRG1 in the proliferation and apoptosis of B-ALL cells both in vivo and in vitro. Mechanistically, BRG1 directly inhibits PPP2R1A, thereby initiating the PI3K/AKT signaling pathway and regulating the expression of many genes related to the cell cycle and apoptosis. Therefore, BRG1 may serve as a valuable marker for assessing the onset of B-ALL in adults and a promising target for treating the disease.

## Results

### BRG1 is overexpressed in newly diagnosed B-ALL patients and is associated with worse outcomes

**Fig. 1:**
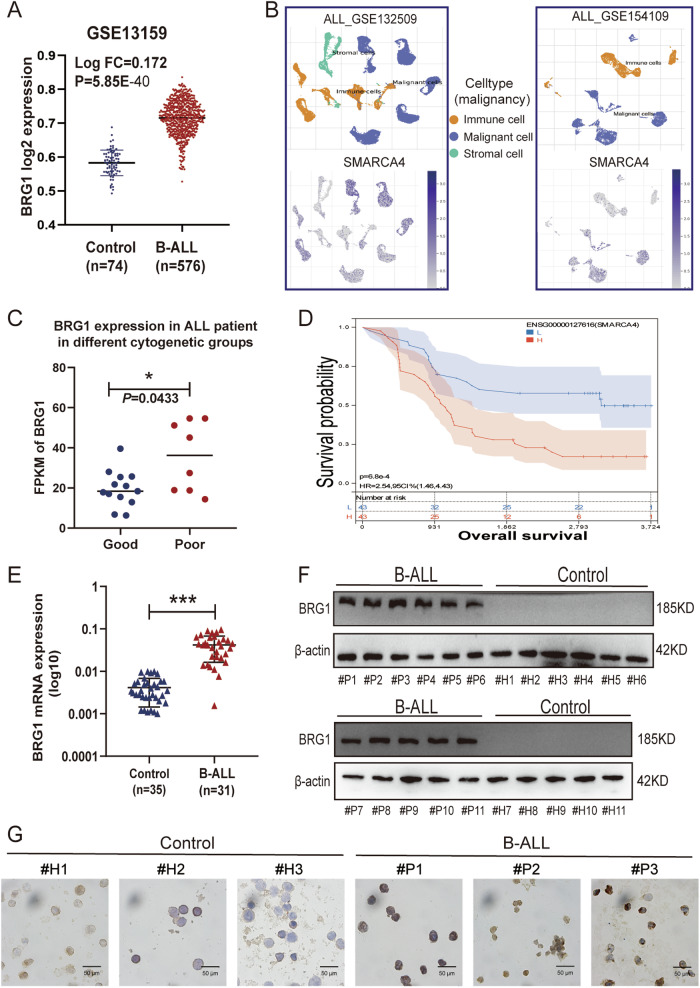
BRG1 is overexpressed in B-ALL patients and is associated with worse outcomes. **A** mRNA expression of SMARCA4 in the GSE13159 dataset (B-ALL, *n* = 576; Control, *n* = 74). Comparative analysis of single-cell sequencing data from the GSE132509 and GSE154109 datasets, with orange representing immune cells, blue representing malignant cells, and green representing stromal cells. **B** Images of BRG1 expression level analysis in immune cells, malignant cells and mesenchymal cells. National Comprehensive Cancer Network (NCCN) Guidelines version 1.2023-defined risk groups were used to divide tumour samples in the TCGA dataset for SMARCA4 gene expression (logPRKM). **C** Scatter-bar plot of mRNA expression of BRG1 in TCGA adult B-ALL dataset aged >18 years (6570 days). **D** Kaplan–Meier survival curve in TARGET dataset. **E** The mRNA expression of BRG1 was analysed via qRT-PCR in healthy control (*n* = 35) and patients with B-ALL (*n* = 31). **F** The expression of BRG1 was detected by immunoblotting in healthy control (#H1-#H11) and patients with B-ALL (#P1-#P11). **G** Representative images of ICC staining of BRG1 in B-ALL samples (#P1-#P3) and healthy control samples (#H1-#H3) (scale bar = 50 μm). (*P < 0.05; ***P < 0.001).

To examine the role of BRG1 in B-ALL, we first analysed its expression in patients with newly diagnosed B-ALL (*n* = 576) and healthy individuals (Control, *n* = 74) in the GSE13159 dataset [20, 21]. The results showed that BRG1 expression was higher in patients with B-ALL than in healthy individuals (*P* < 0.05) (Fig. 1A). Moreover, analysis of BRG1 expression in different cells in the GSE132509 [22] and GSE154109 [23] datasets showed that malignant cells had significantly higher BRG1 expression than normal cells (immune and stromal cells) (Fig. 1B). Subsequently, patients in the TCGA B-ALL cohort were divided into risk groups based on cytogenetic differences to clarify the relationship between BRG1 expression and patient prognosis. The results revealed that BRG1 expression was markedly lower in the good-risk group than in the poor-risk group (*P* = 0.0433) (Fig. 1C). However, considering the limited data available on adult B-ALL patients in the TCGA and GEO database, we further extracted and analyzed the RNA-Seq data of ALL patients aged >18 years (6570 days) in the Therapeutically Applicable Research to Generate Effective Treatments (TARGET) database. The results showed that BRG1 was an independent prognostic factor for B-ALL (Hazard Ratio = 1.50, *P* = 0.01) (Fig. S1A). Consistently, upregulated BRG1 expression was associated with poor survival in patients with B-ALL (Fig. 1D). Meanwhile, we noted that BRG1 expression was higher in the relapse group than in the remission group (Fig. S1B), but no significant difference was observed between the two groups (*P* = 0.08), which was attributed to the limitations of the sample size and the age distribution of the patients. We also analyzed the transcript levels of SMARCA2 (Fig. S1C), the other ATPase that is mutually exclusive with SMARCA4, and no significant difference was seen between the two groups (*P* = 0.9074). This data demonstrated the trend of BRG1 expression in adult B-ALL. To further enhance the data, we enrolled adult B-ALL patients from our own center for the study. The mRNA (Fig. 1E) and protein (Fig. 1F) expression levels of BRG1 were higher in clinical B-ALL bone marrow mononuclear cells (BMMNCs) samples than in healthy BMMNCs (Control) samples collected at our centre (*P* < 0.05). BMMNCs were isolated from these clinical samples for Immunocytochemical (ICC) analysis. The results showed that BRG1 was upregulated in most cells isolated from B-ALL samples (Figs. 1G, S1D). Altogether, these results indicate that BRG1 is abundantly expressed in newly diagnosed B-ALL and is associated with a poor prognosis.

### BRG1 inhibition leads to cell cycle arrest and increased apoptosis in B-ALL in vitro

**Fig. 2:**
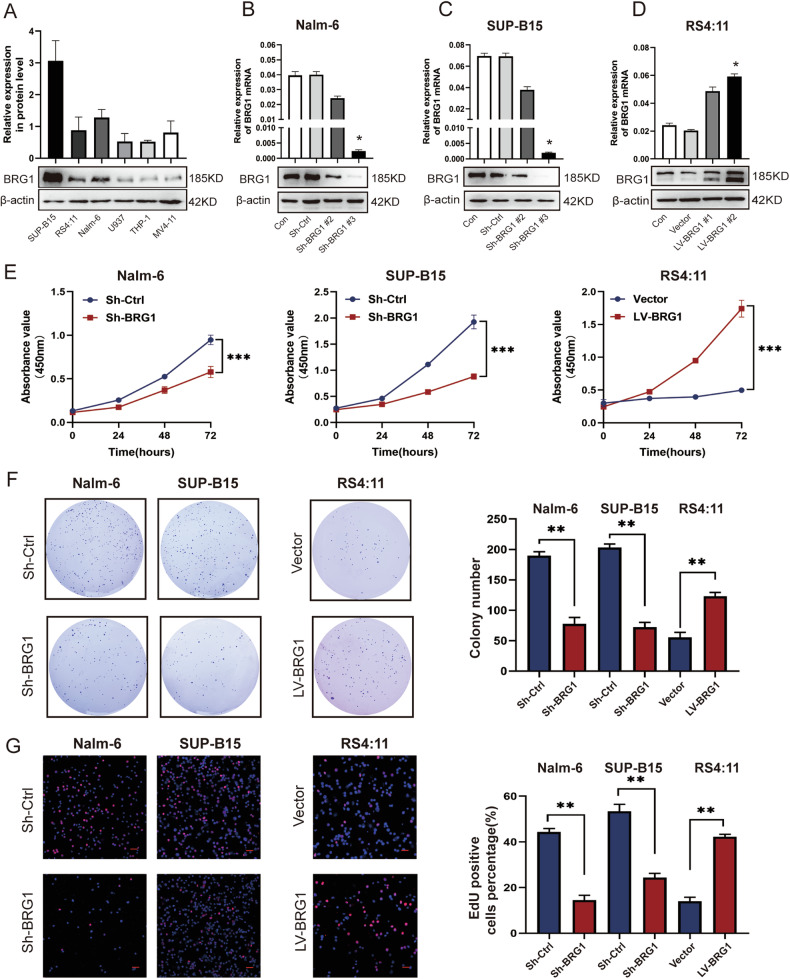
BRG1 promoted the proliferative ability of B-ALL cells in vitro. **A** Three ALL cell lines (SUP-B, RS4:11 and Nalm-6) and three AML cell lines (MV4-11, U937 and THP-1) were analysed via western blotting. Nalm-6 and SUP-B15 cells were transfected with lentiviral vectors encoding puromycin-inducible control ShRNA (Sh-Ctrl) or BRG1 ShRNA (Sh-BRG1), whereas RS4:11 cells were transfected with control (vector) or ectopic BRG1 (LV-BRG1). Puromycin was added to induce shRNA and over-expression virus vector expression for 7 days. Of the three ShRNAs that were used to suppress SMARCA4 expression, SMARCA4#3 was selected for subsequent experiments. Nalm-6 cells (**B**), SUP-B15 cells (**C**) and RS4:11 cells (**D**) were verified via western blotting and qRT-PCR. **E** Cell growth was assessed using the CCK-8 assay at 0, 24, 48, and 72 h. Cells were cultured in 6-well plates using soft agar for 14–18 days. **F** The proliferative ability of BRG1-knockdown or BRG1-overexpressing B-ALL cell lines was assessed using colony-forming assays. **G** Sample micrographs on the left and measurement of EdU incorporation on the right (scale bar = 200 μm). All experiments were repeated three times independently. Data are expressed as the mean ± standard error of the mean (*P < 0.05; **P < 0.01; ***P < 0.001).

**Fig. 3:**
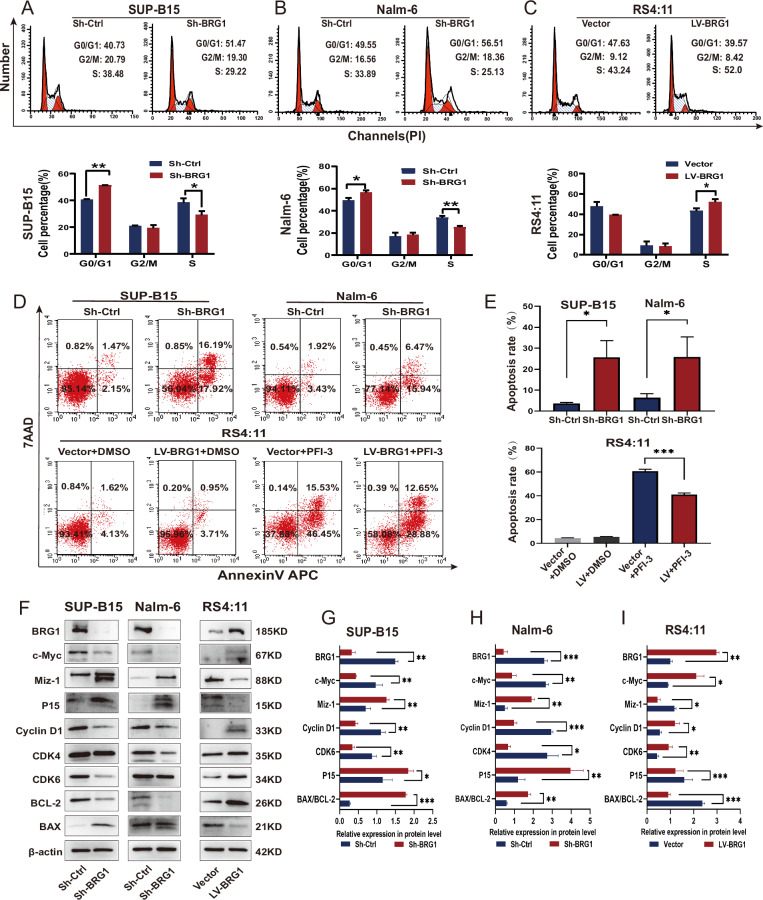
BRG1 enhances cell proliferation and resists apoptosis in B-ALL cells. Cell cycle analyzed by PI labeling. Flow cytometry (top) was used to analyse the cell cycle of BRG1-knockdown (**A**–**B**) or BRG1-overexpressing (**C**) B-ALL cell lines. The bottom image shows cell cycle analysis. LV-BRG1 cells were treated with a vehicle (dimethyl sulfoxide, DMSO) or PFI (100 nM) for 24 h. **D**, **E** Apoptosis of Sh-BRG1, LV-BRG1 and LV-BRG1 with administration cells was analysed via 7-ADD/annexin V labelling. Apoptosis rate is equal to the sum of the frequencies of the two right quadrants (which correspond to early apoptosis (bottom) and late apotosis/necrosis (upper). **F** Western blotting was used to identify markers of proliferation and apoptosis in two BRG1-knockdown and one BRG1-overexpressing B-ALL cell lines. β-actin functioned as the control for loading. **G**–**I** Histogram demonstrating the relative grey values. All experiments were repeated three times independently. Data are expressed as the mean ± standard error of the mean (*P < 0.05; **P < 0.01; ***P < 0.001).

The abovementioned results suggest that BRG1 promotes tumorigenesis in B-ALL. To verify these results, we evaluated the endogenous protein expression of BRG1 in three human B-ALL cell lines (SUP-B15, Nalm-6 and RS4:11) and three human AML cell lines (THP-1, U937 and MV4-11) via western blotting. The results showed that BRG1 expression was higher in B-ALL cells than in AML cells. Furthermore, SUP-B15 and Nalm-6 exhibited higher endogenous BRG1 expression, while RS4:11 showed lower BRG1 expression (Fig. 2A). Based on this fact, lentiviral vectors were used to establish stable SUP-B15 and Nalm-6 cells with silenced BRG1 and RS4:11 cells with ectopic BRG1 expression. Western blotting and quantitative reverse transcription PCR (qRT-PCR) showed that treatment with Sh-BRG1 dramatically reduced BRG1 expression when compared with Sh-Ctrl treatment (Fig. 2B, C). However, the LV-BRG1 group exhibited notably higher expression of BRG1 (Fig. 2D). Cell Counting Kit-8 (CCK-8) assay showed that cell proliferation was significantly reduced in both BRG1-silenced cell lines but enhanced in ectopic BRG1-expressing cells (Fig. 2E). The colony-forming ability of RS4:11/LV-BRG1 cells was considerably high, whereas that of SUP-B15/Sh-BRG1 and Nalm-6/Sh-BRG1 cells was weaker than that of Sh-Ctrl cells (Fig. 2F). Furthermore, EdU incorporation assay was used to examine changes in cell proliferation. Immunofluorescence staining showed that DNA synthesis was remarkably suppressed in both BRG1-silenced cell lines but was enhanced in cells with ectopic expression of BRG1 (Fig. 2G). Additionally, flow cytometry showed that ectopic BRG1 in RS4:11 cells accelerated the progression of the cell cycle into the S phase, whereas BRG1 knockdown in SUP-B15 and Nalm-6 cells induced G0/G1 arrest (Fig. 3A–C). Besides that, flow cytometry was also used to assess whether BRG1 was related to cell apoptosis in B-ALL. The results showed that the apoptotic rate of SUP-B15/Sh-BRG1 and Nalm-6/Sh-BRG1 cells was dramatically higher than that of Sh-Ctrl cells, indicating that high expression of BRG1 strengthened the anti-apoptotic ability of B-ALL cells. To verify these findings, RS4:11/LV-BRG1 cells were treated with PFI-3 (100 nM), a specific inhibitor targeting the bromodomain of SMARCA4 [24]. The results showed that PFI-3 profoundly enhanced the apoptosis of RS4:11/LV-BRG1 cells (Fig. 3D, E). More importantly, immunoblotting results showed that BRG1 silencing decreased the expression of CDK4 (or CDK6), c-Myc, Cyclin D1, BCL-2 and increased the expression of Miz-1, p15 and BAX. In contrast, ectopic expression of BRG1 showed the reverse effect (Fig. 3F–I). These findings suggest that changes in the cell cycle are partly responsible for changes in cell proliferation and that BRG1 stimulates G1/S transition in B-ALL cells to increase cell proliferation. Meanwhile, the reduced BAX/BCL-2 ratio confirmed that BRG1 can induce apoptosis resistance in B-ALL cells.

### BRG1 inhibition suppresses B-ALL cell proliferation and prolongs survival in vivo

**Fig. 4:**
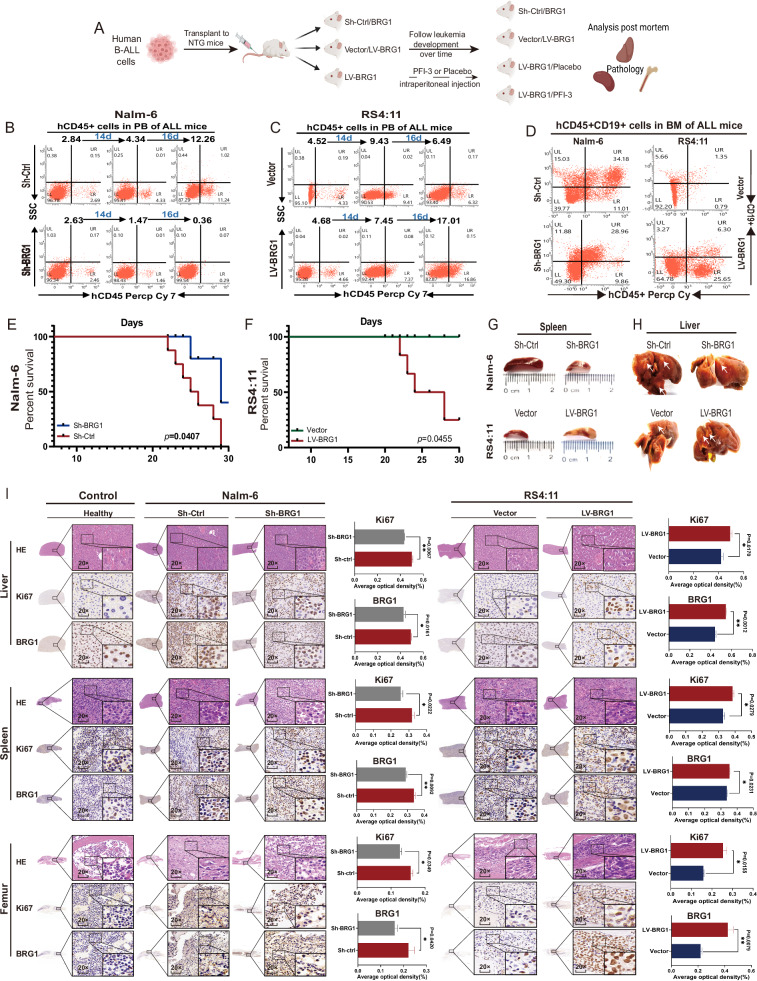
BRG1 facilitates tumor growth in vivo. Five-week-old female NOD-Prkdcscid IL2rgnull (NTG) mice (*n* = 8/group) were administered 1 × 10^7^ Nalm-6/Sh-BRG1 cells, Nalm-6/Sh-Ctrl cells, RS4:11/LV-BRG1 cells or RS4:11/LV-Vector cells via tail vein injection. **A** Schematic diagram of injected cells into NTG mice. The survival of diseased mice was monitored. Peripheral blood (PB) was collected weekly from the mice angular vein and labeled with anti-human CD45+ antibody and analyzed by flow cytometry. Proportion of CD45+ cells in human B-ALL cells in the angular vein of Nalm-6 mice (**B**) and RS4:11 mice (**C**). After the mice were sacrificed, bone marrow (BM) cells were labelled with anti-human CD45+ and CD19+ antibodies. Flow cytometry was used to assess the engraftment of human cells. **D** Proportion of human CD45+ and CD19+ cells in the BM of mice in the groups. **E**, **F** Survival of mice transplanted with two types of samples (*n* = 8 per group). Representative gross illustrations of spleen (**G**) and liver (**H**) metastases in mice injected with cells. White arrows indicate neoplasm invasiveness. **I** H&E staining and IHC (BRG1 and Ki67 expression) results of the liver, spleen and femur in the normal, Nalm-6 and RS4:11 groups. The histogram shows the average proportion of BRG1- and Ki67-positive cells (*P < 0.05; **P <0.01).

The abovementioned results suggest that BRG1 promotes cell proliferation and inhibits apoptosis in B-ALL in vitro. To verify these results, we established xenograft mouse models of B-ALL to assess the potential effects of BRG1 on the growth of B-ALL cells in vivo (Fig. 4A). Compared with Nalm-6/Sh-Ctrl cells, Nalm-6/Sh-BRG1 cells had a lower percentage and total number of human CD45+ leukemia cells in angular vein (Fig. 4B) and CD45 + CD19+ leukemia cells in bone marrow (BM) (Fig. 4D). However, BRG1 overexpression exerted the opposite effect (Fig. 4C, D). Furthermore, BRG1 knockdown prolonged survival and BRG1 overexpression reduced survival in mice with B-ALL (Fig. 4E, F). Consistently, gross organ metastatic foci were fewer in the Sh-BRG1 group than in the Sh-Ctrl group; however, LV-BRG1 mice had more gross organ metastatic foci than LV-Vector mice (Figs. 4G, H, S2E). Hematoxylin-Eosin (H&E) and immunohistochemical (IHC) staining were performed to assess cell proliferation in the liver, spleen and femur of mice (3 mice were randomly selected from each group). H&E staining showed the histological characteristics of the mouse liver, spleen and femur. IHC staining revealed that the expression of Ki-67 was higher in mice injected with BRG1-overexpressing cells and significantly low in mice injected with BRG1-knockdown cells (Fig. 4I). These findings suggest that BRG1 plays an important role in the growth of B-ALL cells.

To facilitate the clinical translation of the abovementioned findings, we examined the outcomes of a placebo (corn oil) or PFI-3 in mouse models injected with RS4:11 cells with ectopic BRG1 expression. The results showed that PFI-3 treatment reduced the percentage and total number of human CD45+ leukemia cells in angular vein (Fig. S2A) and CD45 + CD19+ leukemia cells in BM (Fig. S2B). Notably, survival was better in mice treated with PFI-3 than in control mice (Fig. S2C). Consistently, gross organ metastatic foci were fewer in mice treated with PFI-3 than in mice treated with a placebo (white arrows represent metastatic foci) (Fig. S2D, E) Accordingly, IHC staining showed that treatment with PFI-3 decreased Ki67 expression in mouse organs. These results indicate that pharmaceutical inhibition of BRG1 is beneficial for B-ALL (Fig. S2F).

### c-Myc overexpression is important for the regulatory effects of BRG1 on the B-ALL cell cycle

**Fig. 5:**
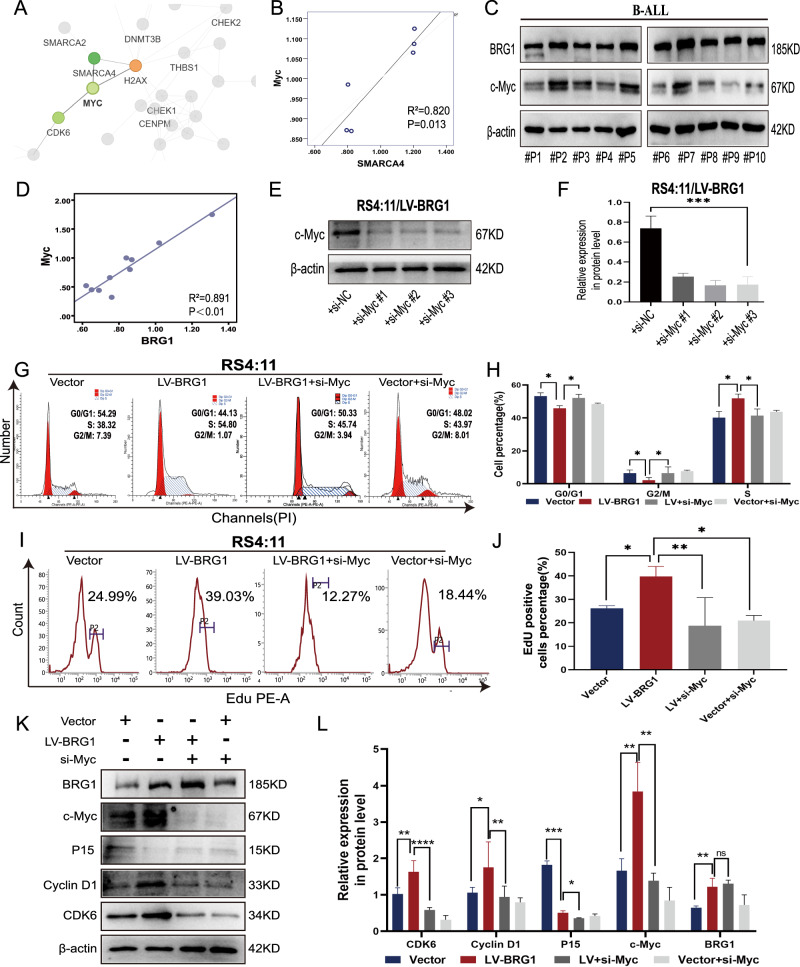
BRG1 and c-Myc cooperate to sustain the growth of B-ALL cells. BRG1-knockdown Nalm-6 cells (Sh-BRG1) and control cells (Sh-Ctrl) were analysed via TMT-labelled proteomics, and 165 differential proteins were acquired. **A** The R software package ‘network D3’ was used to visualise the differential protein–protein interaction network. Red denotes upregulated proteins, and green denotes downregulated proteins. The bigger the multiple of difference, the darker the hue. **B** Correlation between BRG1 and c-Myc. **C** Primary ALL blast cells from 10 patients with B-ALL (#P1-#P10) were analysed via immunoblotting for BRG1 and c-Myc. **D** Correlation between BRG1 and c-Myc in 10 B-ALL samples. β-actin was used as the loading control. The siRNAs and Namipo^TM^ were carefully mixed (1:1) to a final concentration of 50 nM and incubated for 10 min at room temperature. Ectopic BRG1-overexpressing RS4:11 cells (LV-BRG1) were treated with three siRNAs targeted against siRNA–Namipo^TM^ complexes for 72 h, and the silencing efficiency was detected via western blot (**E**). **F** Histogram demonstrating the relative grey values. Ectopic BRG1-expressing RS4:11 cells (LV-BRG1) and control cells (Vector) were treated with siRNA to transiently inhibit c-Myc expression. The cell cycle (**G**) and EdU incorporation (**I**) changes in each group were analysed via flow cytometry. Cell cycle analysis (**H**) and EdU positive cell percentage (**J**) are displayed by in the histograms. **K** Cell cycle-related proteins were detected via western blotting in both LV-BRG1 cells or control cells (Vector) induced by c-Myc siRNA. **L** Histogram demonstrating the relative grey values. Data are expressed as the mean ± standard error of the mean (*P < 0.05; **P < 0.01; ***P < 0.001; ****P < 0.0001; ns not significant).

The abovementioned results indicate that BRG1 promotes cell proliferation and inhibits apoptosis in B-ALL cells. To determine the underlying molecular mechanisms, we performed tandem mass tag (TMT)-labelled proteomics analyses to investigate changes in protein expression in BRG1-knockdown Nalm-6 cells. The results revealed that Myc was downregulated in BRG1-knockdown Nalm-6 cells (*P* = 0.014) (Table S3). The protein–protein interaction (PPI) network of TMT-labelled proteomic indicated that Myc interacted with BRG1 (Fig. 5A) and was positively correlated with BRG1 (R^2^ = 0.820; Fig. 5B). Consistently, the protein expression of c-Myc was notably low in BRG1-silenced SUP-B15 cells but was dramatically high in RS4:11 cells with ectopic BRG1 expression (Fig. 3F). Previous studies have reported that c-Myc is strongly associated with the proliferation of haematologic tumour cells [25–27]. As shown in Fig. 5A, the PPI network suggests that c-Myc is responsible for the regulation of CDK6 and hence affects cell proliferation. Therefore, we hypothesised that c-Myc might be a downstream effector molecule of BRG1 in regulating the proliferation of B-ALL cells. Analysis of clinical samples collected at our centre showed a positive correlation between BRG1 and c-Myc expression in BMMNCs from patients with B-ALL (R^2^ = 0.891; Fig. 5C, D). Furthermore, a small interfering RNA (siRNA) with the highest silencing efficiency was used to silence c-Myc expression in ectopic BRG1-expressing ALL cells (Fig. 5E, F). Flow cytometry showed that silencing of c-Myc suppressed the transition to the S phase of the cell cycle (Fig. 5G, H) and DNA synthesis (Fig. 5I, J) in cells with ectopic expression of BRG1. Consistently, immunoblotting showed that silencing of c-Myc inhibited cell cycle-related proteins (Fig. 5K, L). Altogether, these results indicate that maintaining the endogenous expression of Myc is necessary for BRG1 to regulate the cell cycle in B-ALL.

### BRG1 participates in the regulation of cell cycle-associated proteins by regulating c-Myc expression through PI3K/AKT pathway activity

**Fig. 6:**
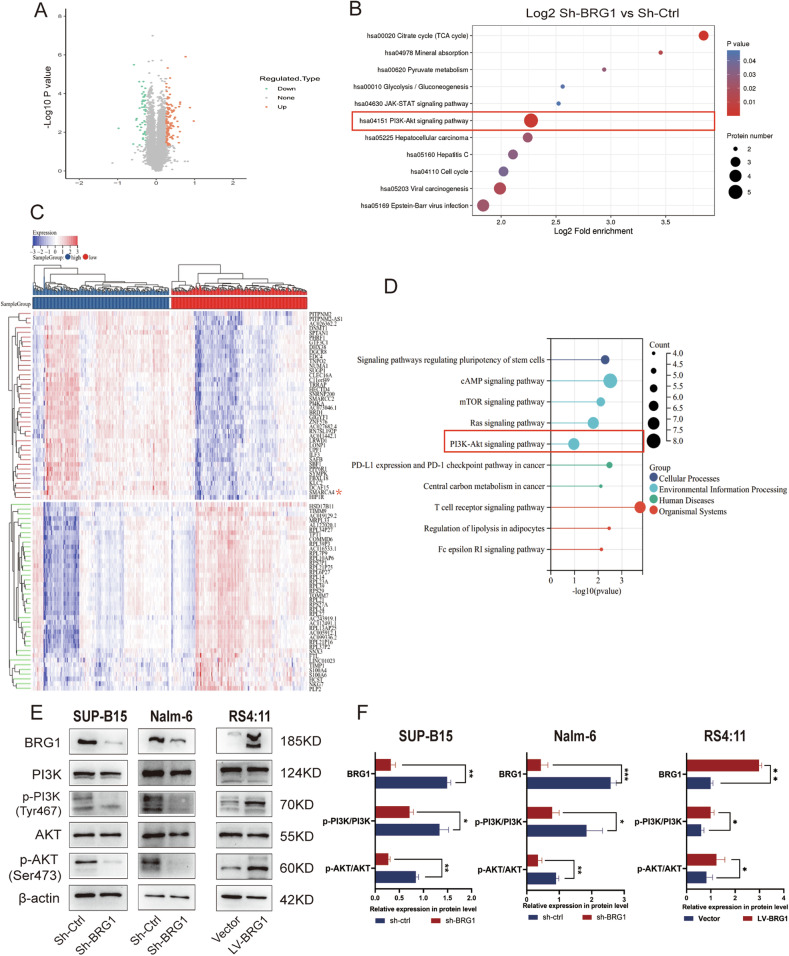
BRG1 regulates cell cycle-associated proteins via the PI3K/AKT pathway. BRG1-knockdown Nalm-6 cells (Sh-BRG1) and control cells (Sh-Ctrl) were analysed via TMT-labelled proteomics, and differential proteins were presented on the volcano plot (**A**). Red represents predicted upregulated proteins (*n* = 114), and green represents downregulated proteins (*n* = 51). **B** KEGG pathway enrichment analysis of differentially expressed proteins. The RNA-seq data of B-ALL patients extracted from the TCGA dataset were categorized into high-expression BRG1 and low-expression BRG1 groups based on the median expression levels of BRG1. **C** Heatmap of differentially expressed genes evaluated based on TCGA (Red asterisks indicates SMARCA4). **D** KEGG pathway enrichment analysis of differential genes based on TCGA. **E** Western blotting was used to identify signaling pathway markers in B-ALL cell lines with either overexpressed or knocked-down BRG1. **F** Histogram demonstrating the relative grey values. Data are expressed as the mean ± standard error of the mean (*P < 0.05; **P < 0.01; ***P < 0.001).

Previous studies have demonstrated that BRG1 regulates c-Myc expression by binding to the c-Myc enhancer in AML [19], and our Chromatin immunoprecipitation (ChIP) followed by next-generation sequencing (ChIP-seq) assay also supported that the binding peak of BRG1 was located at the Myc promoter region (Fig. S5A). However, further assessment of proteomic data showed that 51 genes were downregulated and 114 genes were upregulated after BRG1 knockdown (Fig. 6A; *P* < 0.05) (Table S3). The downregulated genes (Fold change ≤ 1/1.2) were mainly involved in cellular processes and signaling. Kyoto Encyclopedia of Genes and Genomes (KEGG) pathway analysis revealed that the PI3K/AKT signaling pathway was significantly altered (Fig. 6B). Considering the limitations of in vitro experiments, we further analysed the RNA-seq data of patients with ALL with different BRG1 expression levels in TCGA datasets (Fig. 6C). Activation of the PI3K/AKT pathway was more pronounced in patients with B-ALL with high expression of BRG1 (Fig. 6D). Both proteomics and TCGA data analysis suggested a high enrichment of PI3K/AKT signaling pathway, based on which we speculated that BRG1 might regulate the growth and apoptosis of B-ALL cells by regulating the activity of the PI3K/AKT pathway and its downstream molecules. Indeed, although no significant differences in total PI3K and AKT levels were observed, the levels of phosphorylated PI3K^Tyr467^ and Akt^Ser473^ were remarkably lower in the Sh-BRG1 group than in the LV-BRG1 group (Fig. 6E, F). Altogether, these results suggest that BRG1 regulates the PI3K/AKT pathway by enhancing the phosphorylation of PI3K and AKT, consequently modulating proteins related to the cell cycle and apoptosis. BRG1 may regulate the proliferation and apoptosis of B-ALL cells through this mechanism.

### BRG1-mediated anti-apoptosis effects mainly rely on the regulation of BCL-2 and BAX by PI3K/AKT signaling

According to the abovementioned results, silencing of BRG1 enhanced apoptosis and impaired the PI3K/AKT signaling pathway in B-ALL cells. To examine whether BRG1 regulates B-ALL cell apoptosis through the PI3K/AKT signaling pathway, specific inhibitors were used to block relevant signaling pathways to observe changes in downstream molecules. RS4:11/LV-BRG1 cells and their control cells were treated with Ly294002 (an inhibitor of the PI3K/AKT signaling pathway) for 24 h. Treatment with Ly294002 (Fig. S3A, C) readily caused significant apoptosis of ectopic-BRG1-expressing RS4:11 cells, suggesting that the anti-apoptosis effect induced by upregulated BRG1 can be partially eliminated by suppressing PI3K specifically. However, treatment with Ly294002 significantly enhanced apoptosis in RS4:11/LV-Vector cells when compared with RS/LV-BRG1 cells. Moreover, western blotting showed that treatment with Ly294002 partially restored BAX expression and decreased p-PI3K^Tyr467^, p-Akt^Ser473^ and BCL-2 expression in RS4:11 cells with ectopic expression of BRG1 (Fig. S3B, D). Altogether, these results indicate that BRG1-induced anti-apoptosis effects on B-ALL cells are mainly regulated by the PI3K/AKT signaling pathway. In addition, these results suggest the existence of other mechanisms through which BRG1 regulates BCL-2 expression.

### BRG1 suppresses PPP2R1A expression by occupying its transcriptional activation site, thereby activating PI3K/AKT signaling

**Fig. 7:**
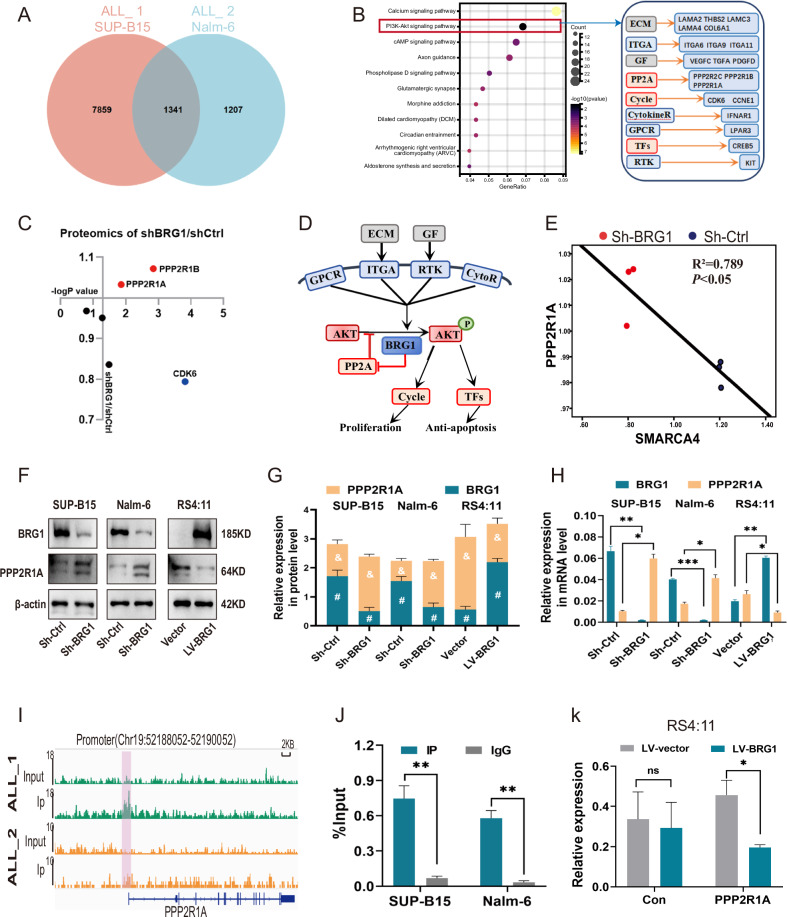
BRG1 suppresses PPP2R1A expression by occupying its transcriptional activation site, thereby activating PI3K/AKT signaling. **A** ChIP-Seq was performed on SUP-B15 (ALL_1) and Nalm-6 (ALL_2) cells, and Venn diagram showing the gene overlap analysis between the ChIP-seq data of the two cell lines. **B** KEGG analysis was performed on the fitted gene clusters, in which the PI3K–AKT signaling pathway was significantly affected, and genes involved in the regulation of the pathway were categorised and analysed, with the grey section representing genes related to extracellular components, the blue section representing genes localised in the cell membrane, and the red section representing genes localised in the cytoplasm. **C** The effect of BRG1 silencing on the products encoded by genes in the cytoplasm in plot B was analysed in the proteomic data, which showed that the expression of PPP2R1A and PPP2R1B was elevated and the expression of CDK6 was decreased. **D** Schematic diagram of the regulation of PP2A and PI3K–AKT signaling pathway by BRG1. Given that the results of ChIP-Seq showed that the site of binding of BRG1 to the PPP2R1B is on the intron and the binding site to PPP2R1A is on the promoter, (**E**) the correlation between PPP2R1A and BRG1 (SMARCA4) was analysed at the protein level. **F** The expression of PPP2R1A in three cell lines with differential expression of BRG1 was evaluated via western blotting. **G** Grey value analysis of the protein expression of plot F. # represents statistical differences in BRG1 expression between groups, & represents statistical differences in PPP2R1A expression between groups. **H** The mRNA expression of PPP2R1A in three cell lines with differential expression of BRG1 was detected by qRT-PCR. **I** ChIP-seq profiling showed the ChIP-seq signal for BRG1 at the genomic loci of PPP2R1A in ALL_1 (-log10(*p*-value) = 7.13) and ALL_2 (-log10(*p*-value) = 3.2). (Red marker indicates PPP2R1A promoter region: Chr19:52188052-52190052). **J** ChIP-qPCR was used to detect the binding of BRG1 to the PPP2R1A promoter in SUP-B15 and Nalm-6 cells. A dual luciferase plasmid overexpressing PPP2R1A was constructed and transfected with it into BRG1-overexpressing RS4:11 cells (LV-BRG1) and control cells (LV-Vector). **K** Histogram showing relative fluorescence intensity of groups. (*, & and #, *P* < 0.05; **P < 0.01; ***P < 0.001; -log10(*p*-value)>1.3, *P* < 0.05; ns not significant).

The abovementioned results validate that activation of PI3K/AKT signaling is essential for the oncogenic effects of BRG1. We next explained the activation mechanism by performing ChIP-seq of BRG1 (Fig. S4). Intersection of the gene sets of the two cell lines (SUP-B15 and Nalm-6) obtained from ChIP-Seq and KEGG analysis revealed significant changes in the PI3K/AKT signaling pathway (Fig. 7A, B). This signaling pathway is located upstream of c-Myc and can regulate its expression [28, 29]. On integrating the results of proteomic analysis and ChIP-seq, we found some genes that can influence the activity of the PI3K/AKT signaling pathway. Correlation analysis of these genes showed that BRG1 was negatively associated with PPP2R1A and PPP2R1B and positively associated with CDK6 (Fig. 7C). Although changes in CDK6 expression were observed in immunoblotting experiments, not all cells were affected (Fig. 3F). Meanwhile, our ChIP-seq data indicated that the binding peak of BRG1 was located in the promoter region of PPP2R1A (Figs. 7I, S5E), not PPP2R1B (Fig. S5B). PPP2R1A has been reported to inhibit the phosphorylation of AKT [30, 31]. Consistently, proteomic data showed that PPP2R1A was negatively correlated with both BRG1 (R^2^ = 0.789) (Fig. 7E) and Myc (R^2^ = 0.601) (Fig. S5D). Altogether, these results suggest that BRG1 inhibits PPP2R1A expression and hence activates the PI3K/AKT signaling pathway (Fig. 7D). This phenomenon was validated in the three B-ALL cell lines with altered BRG1 expression (Fig. 7F–H). Furthermore, ChIP-seq results were used to identify the mechanisms through which BRG1 inhibits PPP2R1A. Remarkably, BRG1 possesses a high binding affinity for the ACTAAAAATACA sequence at the PPP2R1A promoter site (Figs. 7I, S5C), which can bind to multiple transcriptional activators such as Mef2c. Moreover, ChIP-qPCR further verified that BRG1 can bind to the promoter region of PPP2R1A (Fig. 7J). This finding suggests that BRG1 suppresses PPP2R1A expression possibly by occupying transcriptionally activated sites. Therefore, we designed a dual-luciferase reporter plasmid encoding the promoter region of the PPP2R1A gene and transfected it into ectopic BRG1-expressing RS4:11 cells. The results showed that the mRNA expression of PPP2R1A was significantly reduced (Fig. 7K).

## Discussion

**Fig. 8:**
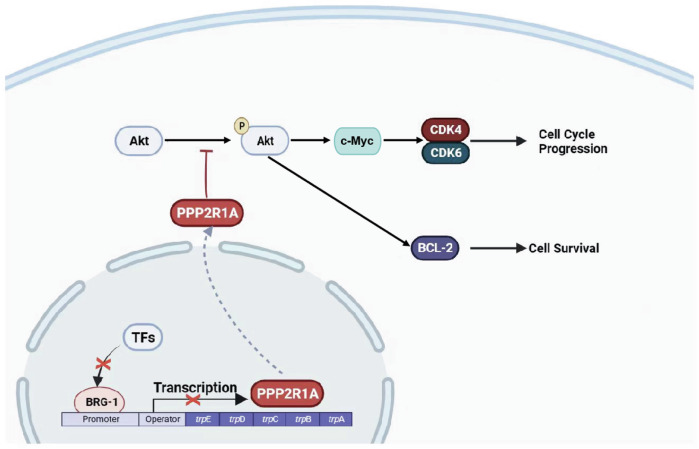
BRG1 promotes progression of B-cell acute lymphoblastic leukemia by disrupting PPP2R1A transcription. Schematic model showing that BRG1 regulates the downstream PI3K/AKT signaling pathway and its target genes by inhibiting the transcription of PPP2R1A.

Although numerous clinical trials have been conducted and novel therapies have been reported in the past decade, the survival and outcome of adult patients with ALL, particularly those above the age of 55 years, remains poor [32, 33]. Therefore, it is necessary to elucidate the mechanisms or specific molecules underlying the pathogenesis of ALL and develop new targeted therapies, which may hold significant promise in achieving deeper first remissions. This study revealed that BRG1 was highly expressed in adult patients with primary B-ALL and was correlated with a worse prognosis. Loss- and gain-of-function experiments demonstrated that ectopic expression of BRG1 enhanced cell proliferation and inhibited apoptosis in B-ALL (Fig. 8). Consistently, BRG1 inhibition and PFI-3 administration suppressed tumorigenesis and enhanced the elimination of CD45 + CD19+ leukemia cells in cell-derived xenograft (CDX) mouse models of B-ALL, suggesting that suppression of BRG1 represents an effective treatment strategy for B-ALL.

One of two mutually exclusive catalytic subunits, SMARCA4 or SMARCA2 (also known as BRG1 or BRM, respectively), is present in SWI/SNF complexes. These subunits interact with different families of transcription factors (TFs) to provide the complexes with specific functional outputs. In addition, they possess DNA-stimulated ATPase activity [34]. BRG1 ATPase is a unique site of assembly for SWI/SNF-like BAF complexes produced in leukemia, whereas BRM (encoded by SMARCA2) is the primary ATPase expressed in quiescent long-term repopulating haematopoietic stem cells (HSCs) [18]. In this study, adult patients with primary B-ALL were found to have higher BRG1 expression than healthy individuals.

Notably, BRG1 is found to be altered in approximately 5–7% of all human malignancies and modulates the growth, division and differentiation of cancer cells [13]. BRG1 acts as a tumour suppressor [35] or a pro-oncogene [36, 37], which depends on the different TFs that BRG1 binds to [13, 38]. This study showed that maintaining the expression of the proto-oncogene Myc was necessary for BRG1 to regulate the proliferation of B-ALL cells, which is consistent with the regulation in AML [19]. More importantly, proteomic analysis, RNA interference and gene inhibition indicated that BRG1 promoted the development of B-ALL by activating the PI3K/AKT signaling pathway, thereby regulating the expression of Myc and apoptosis-related proteins.

PI3K/AKT is one of the most commonly dysregulated signaling pathways in cancer, which is essential for the development and growth of tumours. Notably, previous studies have reported constitutive PI3K/AKT activation in childhood and adult B-ALL [39, 40]; however, the mechanisms underlying this activation remain unclear. In this study, ChIP-seq and dual-luciferase reporter assay revealed that BRG1-mediated inhibition of PPP2R1A was necessary for its regulatory effects on AKT phosphorylation in B-ALL. The scaffold component A-alpha of protein phosphatase 2 A (PP2A), a heterogeneous and abundant phosphatase that modulates several signaling pathways, is encoded by PPP2R1A [41]. In particular, PP2A-A functions as a structural assembly to support the catalytic subunit and enable its interaction with other substrates and the regulatory subunit, suggesting that the A subunits regulate PP2A holoenzyme composition and stabilise its activity [42, 43]. As a tumour suppressor, PP2A blocks the PI3K/AKT signaling pathway by preventing AKT phosphorylation [44, 45]. Given that BRG1 occupies the activation transcriptional site of PPP2R1A and inhibits its expression, we speculate that BRG1 overexpression inhibits PP2A expression by suppressing PPP2R1A transcription, which activates PI3K/AKT signaling to promote the proliferation of B-ALL cells and inhibit apoptosis. Activation of PI3K/AKT by BRG1 has been reported in other cancers [46], but the regulatory effects of BRG1 on PI3K/AKT signaling vary across tumours [47].

Based on the findings of this study, BRG1 is a poor prognostic factor and a promising therapeutic target for B-ALL. Notably, Proteolysis-targeting chimaeras (PROTACs)-induced knockdown of SMARCA4 exhibits pronounced anti-proliferative effects in AML cell lines [48], indicating that targeted degradation of BRG1 is a possible SMARCA4-dependent treatment strategy for cancer. However, B-ALL is a highly heterogeneous disease, and the cell lines selected in this study may not accurately represent the complexity of the regulatory mechanisms of the entire population of B-ALL. Numerous deeper studies, such as involving the patient blast cells and the specific subtypes of B-ALL, remain to be performed.

## Materials and methods

### Patients and clinical samples

All patients were newly diagnosed with B-ALL older than 18 years old confirmed by Morphology, immunology, cytogenetics and molecular biology at the Department of Hematology, Affiliated Hospital of Guizhou Medical University. Table S1 displays the clinical data of the samples. A total of 31 patients with a first diagnosis of B-ALL and 35 healthy individuals were included in this study. Ficoll (Solarbio) extracted BMMNCs derived from B-ALL patients and healthy individuals, which were then utilized to extract proteins or mRNA.

### Cell lines

The cell lines used in this experiment, which included three human B-ALL cell lines (RS4:11, SUP-B15, Nalm-6) and three human AML cell lines (THP-1, U937, MV4-11), were all obtained from the laboratory of the Guizhou Hematopoietic Stem Cell Transplantation Center. All of the cell lines were routinely tested for mycoplasma contamination and verified by short tandem repeat mapping. Liquid nitrogen was used to grow and store each cell line. Following resuscitation, cells were grown at 37 °C and 5% CO_2_ humidity in RPMI-1640 media (Bio-Channel) supplemented with 10% fetal bovine serum (SORFA), 100 units/mL of penicillin, and 100 mg/mL of streptomycin (Coolber).

### Proteomics

BRG1-silenced Nalm-6 cells (sh-BRG1) and their control (sh-Ctrl) were collected (three samples were prepared for each group) and sent to Jingjie PTM BioLabs (Hangzhou, China) for Tandem Mass Tags (TMT)-labelled proteomics analysis. Detailed methodology is provided in the supplementary materials and methods. The proteomics data have been deposited to the ProteomeXchange Consortium (https://proteomecentral.proteomexchange.org/) via the iProX partner repository with the dataset identifier PXD047488.

### Chromatin immunoprecipitation (ChIP) followed by next-generation sequencing (ChIP-seq) assay

SUP-B15 and Nalm-6 cells in logarithmic phase of growth were collected and washed twice with pre-cooled PBS, and then assayed by ChIP-seq. The ChIP-seq data generated and analysed in this article have been uploaded to GEO (https://www.ncbi.nlm.nih.gov/) under accession number GSE249559. Detailed methodology is provided in the supplementary materials and methods.

### Dual-luciferase reporter assay

After 48 h, the cells co-cultured with plasmids were collected, and the activities of Renilla and firefly luciferases were evaluated using the Dual Lumi^TM^ Reporter Assay System Kit (Beyotime) according to the manufacturer’s instructions. Fluorescence intensity detected by VARIOSKAN LUX (Thermo Fisher Scientific). The activity of firefly luciferase was calibrated against that of Renilla luciferase.

### Cell-derived xenograft (CDX) model

The NOD-Prkdcscid IL2rgnull (NTG) female mice, estimated to be 4–6 weeks old, were acquired from Sibeifu (Beijing, China) Laboratory Animal Technology and were raised in Specific Pathogen Free (SPF) environments. Detailed methodology is provided in the supplementary materials and methods.

Please find additional methods and data analysis methods covered in this article in “Supplementary Materials and Methods”.

### Supplementary information

Supplemental materials and methods

Legends for supplementary figures and tables

Supplementary Figure 1-5

Supplementary Table 1

Supplementary Table 2

Supplementary Table 3

Original western blots

## Data Availability

The datasets presented in this study can be found in online repositories. The names of the repository/repositories and accession number(s) can be found in the article/Supplementary Materials. Further inquiries can be directed to the corresponding author.
