# Supplementary material for: BRG1 promotes progression of B-cell acute lymphoblastic leukemia by disrupting PPP2R1A transcription
Source: Cell Death Dis. 2024 Aug 26;15(8):621. doi: 10.1038/s41419-024-06996-w (PMC11347705; doi:10.1038/s41419-024-06996-w)
Supplement: Supplementary file 1 — Supplemental materials and methods [file 41419_2024_6996_MOESM1_ESM.docx]

**Supplemental Materials and Methods**

**Human data retrieval and analysis**

The data set covered in this article was obtained from the Cancer Genome Atlas (TCGA) Research Network ( [https://www.cancer.gov/tcga](https://www.cancer.gov/tcga)), the Gene Expression Omnibus (GEO: https://www.ncbi.nlm.nih.gov/) and the Therapeutically Applicable Research to Generate Effective Treatments (TARGET: <https://ocg.cancer.gov/programs/target).>

Data were preprocessed using batch normalization and centralized processing. According to the NCCN guidelines, we divided the ALL samples into Good and Poor groups and plotted the expression levels of BRG1 in these three groups. High and low expression groups were distinguished based on the median expression level of BRG1. Differentially expressed genes between the high and low expression groups were analyzed using the Limma software package (version 3.40.6), and adjusted *P* < 0.05 and | logFC | ≥ 1.3 were used as thresholds to select differentially expressed genes. Volcano plots were used to show the changes of all genes in the BRG1 high and low expression groups, and heatmaps to show the top 40 differentially expressed genes in the up- and down-regulated groups. Enrichment analysis was performed using the R package clusterProfiler (version 3.14.3) to obtain results of gene set enrichment where *P* < 0.05 was considered statistically significant.

The Tumour Immunity Single Cell Hub (TISCH, http://tisch.comp-genomics.org/home/) provides detailed cell type annotations at the single-cell level, and the TISCH database was used to analytically characterise the expression levels of the BRG1 gene at the single-cell level in patients with ALL, in order to explore in-depth the characteristics of the tumour microenvironment.

**Lentiviral transduction**

Human SMARCA4 overexpression clone lentiviral particles (LV-BRG1) and lentiviral vectors expressing puromycin-inducible BRG1 shRNAs (sh-BRG1) were purchased from Genechem Co., Ltd. (Shanghai, China). All shRNA sequences are listed in Table S2. Stable cell lines with BRG1 overexpression or knockdown were established according to the manufacturer’s instructions. After 5 days of culture in RPMI-1640 medium (Bio-Channel, Naijing, China) supplemented with 10% FBS (SORFA, Beijing, China), stable cell lines with BRG1 overexpression or knockdown were selected and treated with 2–4 μg/mL of puromycin (MedChemExpress, Shanghai, China) for 7 days. qRT-PCR and western blotting were used to assess the overexpression and knockdown efficiency.

**Cell viability**

Cell counting kit-8 (Topscience, Shanghai, China) was used to assess cell viability according to the manufacturer’s instructions. Briefly, cells were seeded in 96-well plates at a density of 3000–5000 cells per well. Subsequently, 10 μL of CCK-8 reagent was added to 100 μL of medium in each well, and the cells were incubated for 1.5 hours at 37°C. Absorbance was measured at 450 nm (A450) on a microplate reader (Tecan, Mechelen, Belgium). All experiments were performed in triplicate.

**5-ethynyl-2´-deoxyuridine incorporation assay**

The Cell-LightTM EdU staining kit (RiboBio, Guangzhou, China) was used to assess cell proliferation according to the manufacturer’s instructions. Images were captured at 10× magnification using an Olympus microscope (Tokyo, Japan). The Image J software was used to calculate the ratio of red-fluorescent EdU-positive cells to blue-fluorescent Hoechst-stained cells. In addition, flow cytometry was used to quantify EdU-positive cells. All experiments were performed in triplicate.

**Soft-agar colony formation assay**

For colony formation assay, 2% agarose (low-melting gel) (Solarbio, Beijing, China) was prepared in sterile water and autoclaved. A culture system was established with two layers of gels in a 6-well plate. In particular, 1 mL of a medium containing 15% FBS and 0.5 agarosewere added to the bottom layer, whereas 1 mL of a medium containing 15% FBS, 0.35% agarose and 350–500 cells were added to the top layer. After 14–18 days of culture, the cells were washed with PBS and stained with 0.05% crystal violet. Subsequently, cell colonies were counted using the ImageJ software (NIH, USA). The experiment was repeated at least three times independently. All experiments were performed in triplicate.

**Immunocytochemical (ICC) and immunohistochemical (IHC) staining**

ICC and IHC analyses were performed using anti-BRG1 (HUABIO, ET1611-85, Hangzhou, China) and anti-Ki67 (Absin, abs149852, Shanghai, China) antibodies to assess protein expression. The Image J software was used to score the Integratedoption Density (IOD). To determine the Average Optial Density (AOD), we divided the area by the IOD.

**Cell cycle analysis**

The Cell Cycle Assay Kit (keygentec, Nanjing, China) was used to assess cell cycle according to the manufacturer’s instructions. Briefly, human B-ALL cells were washed twice with ice-cold PBS buffer, fixed with ice-cold 70% ethanol at 4°C overnight and stained with 50 μg/mL of propidium iodide (PI) (keygentec, Nanjing, China). Subsequently, the DNA content of cells in each group was evaluated using flow cytometry (FACS Calibur, Becton Dickinson). Cell cycle analysis did not involve synchronisation, and each test was run three times. All experiments were performed in triplicate.

**Apoptosis assay**

To assess apoptosis, cells were stained with Annexin V-APC and 7-AAD according to the manufacturer’s instructions (MULTI SCIENCE, Hangzhou, China). Flow cytometry and the Cell Quest software (BD Biosciences, San Jose, CA, USA) were used to quantify apoptotic cells. Furthermore, RS4:11/LV-BRG1 and RS4:11/Vector cells were seeded in 24-well plates and treated with PFI-3 (100 nM) (MedChemExpress, Shanghai, China) or DMSO (control) (Solarbio, Beijing, China). After 24 hours of incubation, the cells were harvested and apoptosis was assessed using flow cytometry. All experiments were performed in triplicate.

**RNA extraction and quantitative reverse transcription PCR (qRT-PCR)**

Total RNA was extracted from cells using Trizol (Invitrogen, Carlsbad, CA, USA) and reverse transcribed to cDNA using RT Master Mix for qPCR II (MedChemExpress, Shanghai, China) according to the manufacturer’s instructions. Subsequently, qPCR was performed on the Applied Biosystems 7500 Fast Real-Time RCR System (Applied Biosystems, Foster City, CA, USA) using the SYBR Green qPCR Master Mix (Universal) (MedChemExpress, Shanghai, China). The relative expression of target genes was evaluated using the 2^-△△Ct^ method. The primer sequences used for PCR are listed in Table S2. The experiment was performed three times independently.

**Western blotting**

Total proteins were extracted from all B-ALL cells and clinical samples using RIPA buffer (Solarbio, Beijing, China). The extracted proteins were quantified using a BCA protein assay kit (Pierce, Hercules, CA, USA). Subsequently, 30 μg of protein from each group was separated on 8–12% sodium dodecyl sulphate–polyacrylamide gels and transferred to PVDF membranes. The membranes were blocked with 5% non-fat dry milk for 2 hours and incubated with primary antibodies overnight. Antibodies used are as follows (used at 1:1,000 unless noted otherwise): 1:5000 β-actin (Proteintech, 20536-1-AP); BRG1 (HUABIO, ET1611-85); P15 (Immunoway, YT3492); Miz-1 (Immunoway, YN0110); c-Myc (HUABIO, RT1149); CDK4 (Immunoway, YT5198); CDK6 (Immunoway, YT5884); Cyclin D1 (HUABIO, ET1601-31); BCL-2 (Immunoway, YT0470); BAX (Immunoway, YT0455); 1:500 phospho-Ser473 AKT (Immunoway, YP0006); total AKT (Immunoway, YT0185); 1:500 phospho-Tyr467/199 PI3-Kinase p85/p55 (Immunoway, YP0224); total PI3 Kinase P85α (Immunoway, YM3503); PPP2R1A (Immunoway, YN0090). The following day, the membranes were incubated with horseradish peroxidase-conjugated anti-rabbit IgG (1:10000, Proteintech, PR30011) or anti-mouse IgG antibodies (1:10000, Proteintech, PR30012, ) for 1 hour at room temperature. Subsequently, protein bands were visualised on the Tanon 4200 automatic chemiluminescence image analysis system (Tanon, Shanghai, China) using the UltraSignal ECL Western Blotting Detection Reagent (4A Biotech, Suzhou, China). The intensity of protein bands was quantified using the ImageJ software. The cell line experiment were performed in triplicate.

**Cell-derived xenograft (CDX) model**

The Guizhou Medical University Animal Care & Use Committee approved all experimental animal operations (Approval No. 2201138), and the animals were reared in Specific Pathogen Free (SPF) environments. The female NOD-Prkdcscid IL2rgnull (NTG) mice, estimated to be 4-6 weeks old, were acquired from Sibeifu (Beijing, China) Laboratory Animal Technology.

Cell-derived xenograft (CDX) model: Five-week-old female NTG mice (n = 8) were administered 1 × 10^7^ Nalm-6/sh-BRG1 cells, Nalm-6/sh-Ctrl cells, RS4:11/LV-BRG1 cells or RS4:11/vector cells via tail vein injection.

PFI treatment: Configure the working solution as follows: a. Dilute PFI-3 powder with DMSO to form a 70 mM drug solution and store it at -20℃; b. Before injection, take out the PFI-3 drug solution and dilute it 10-fold for intraperitoneal (i.p) injection. Five-week-old female NTG mice (n = 8/group) were administered 1 × 10^7^ RS4:11/LV-BRG1 cells via tail vein injection. Upon verification of leukaemia cell engraftment in mouse peripheral blood (>0.5% human CD45+ cells), the mice were treated with either PFI-3 (10 mg/kg/d) or corn oil (control) for 1 week.

After mouse models of leukaemia were established, mouse survival and the number of human leukaemia cells in mouse peripheral blood (human CD45+ cells) were monitored twice a week. After the mice were sacrificed, bone marrow cells were labelled with anti-human CD19 APC (BD, #340437) and anti-human CD45 PerCP (BD, #664934). Flow cytometry was used to assess the engraftment of human cells. In addition, immunohistochemical (IHC) and haematoxylin and eosin (H&E) staining were performed using antibodies against BRG1 (HUABIO, ET1611-85) and Ki-67 (Absin, abs149852) to validate the presence of cancer cells in tumour tissues (3 mice were randomly selected from each group).

**Proteomics**

BRG1-silenced Nalm-6 cells (sh-BRG1) and their control (sh-Ctrl) were collected (three samples were prepared for each group) and sent to Jingjie PTM BioLabs (Hangzhou, China) for Tandem Mass Tags (TMT)-labelled proteomics analysis. The proteomics data have been deposited to the ProteomeXchange Consortium ( [http://proteomecentral. proteomexchange.org](http://proteomecentral.proteomexchange.org/)) via the iProX partner repository with the dataset identifier [PXD047488](http://proteomecentral.proteomexchange.org/cgi/GetDataset?ID=PXD047488).

Experimental Procedures: Nalm-6/Sh-ctrl and Nalm-6/Sh-BRG1 cells were sonicated three times on ice using a high intensity ultrasonic processor (Scientz) in lysis buffer (8 M urea, 1% Protease Inhibitor Cocktail). The remaining debris was removed by centrifugation at 12,000 g at 4 °C for 10 min. Then, the supernatant was collected and the protein concentration was determined with BCA kit according to the manufacturer’s instructions. For digestion, the protein solution was reduced with 5 mM dithiothreitol for 30 min at 56 °C and alkylated with 11 mM iodoacetamide for 15 min at room temperature in darkness. The protein sample was then diluted by adding 100 mM TEAB to urea concentration less than 2M. Finally, trypsin was added at 1:50 trypsin-to-protein mass ratio for the first digestion overnight and 1:100 trypsin-to-protein mass ratio for a second 4 h-digestion. After trypsin digestion, peptide was desalted by Strata X C18 SPE column (Phenomenex) and vacuum-dried. Peptide was reconstituted in 0.5 M TEAB and processed according to the manufacturer’s protocol for TMT kit. Briefly, one unit of TMT reagent were thawed and reconstituted in acetonitrile. The peptide mixtures were then incubated for 2 h at room temperature and pooled, desalted and dried by vacuum centrifugation. The tryptic peptides were dissolved in solvent A (0.1% formic acid, 2% acetonitrile), directly loaded onto a home-made reversed-phase analytical column (25-cm length, 100 μm i.d.). Peptides were separated with a gradient from 7% to 25% solvent B (0.1% formic acid in 90% acetonitrile) over 24 min, 25% to 35% in 8 min and climbing to 80% in 4 min then holding at 80% for the last 4 min, all at a constant flowrate of 450 nL/min on an EASY-nLC 1000 UPLC system (Thermo Fisher Scientific).The separated peptides were analyzed in Q ExactiveTM Plus (Thermo Fisher Scientific) with a nano-electrospray ion source. The electrospray voltage applied was 2.0 kV. The full MS scan resolution was set to 75,000 for a scan range of 400–1500 m/z. Up to 20 most abundant precursors were then selected for further MS/MS analyses with 30 s dynamic exclusion. The HCD fragmentation was performed at a normalized collision energy (NCE) of 28%. The fragments were detected in the Orbitrap at a resolution of 17,500. Fixed first mass was set as 100 m/z. Automatic gain control (AGC) target was set at 5E4, with an intensity threshold of 7.8E4 and a maximum injection time of 64 ms.

Data Processing

a. Screening of differential proteins：In order to determine the significance of the differences, the peptide relative quantification values of each protein in the comparison group were subjected to a t-test, and the corresponding p-value was calculated as an indicator of significance, with a default *p* < 0.05. In order for the test data to conform to the normal distribution required by the t-test. Before the test, the relative quantitative values of peptides need to be Log2 log-transformed. The formula is as follows: where U denotes peptide relative quantitative value and j denotes peptide.

*P_k_=T.test(Log2(U_Aj_),Log(U_Bj_),jεk)*

b. Enrichment of pathway analysis: KEGG database was used to identify enriched pathways by a two-tailed Fisher’s exact test to test the enrichment of the differentially expressed protein against all identified proteins. The pathway with a corrected p-value < 0.05 was considered significant. These pathways were classified into hierarchical categories according to the KEGG website.

c. Protein-protein Interaction Network: All differentially expressed protein database accession or sequence were searched against the STRING database version 11.0 for protein-protein interactions. Only interactions between the proteins belonging to the searched data set were selected, thereby excluding external candidates. STRING defines a metric called “confidence score” to define interaction confidence; we fetched all interactions that had a confidence score ≥ 0.7 (high confidence). Interaction network form STRING was visualized in R package “networkD3”.

**Small interfering RNAs**

Three small interfering RNAs (siRNAs) targeting Myc (si-Myc-1, 2 and 3) and a negative-control (NC) siRNA that had no specific target were generated by TranSheepBio (Shanghai, China). The primer sequences for siRNAs targeting Myc are listed in Table S2. The siRNAs and NamipoTM (TranSheepBio, Shanghai, China) were carefully mixed (1:1) to a final concentration of 50 nM and incubated for 10 minutes at room temperature. These siRNA–Namipo^TM^ complexes were transfected into RS4:11/Vector and RS4:11/LV-BRG1 cells, which were cultured at 37°C in a CO_2_ incubator for 72 hours. Subsequently, western blotting was used to assess the effectiveness of c-Myc knockdown.

**Chromatin immunoprecipitation (ChIP) followed by next-generation sequencing (ChIP-seq) assay and ChIP-qPCR**

SUP-B15 and Nalm-6 cells in logarithmic phase of growth were collected and washed twice with pre-cooled PBS, and then subjected to ChIP-seq assay. The ChIP-seq data generated and analysed in this article have been uploaded to GEO (https://www.ncbi.nlm.nih.gov/) under accession number GSE249559.

Experimental Procedures: SUP-B15 (ALL_1) and Nalm-6 (ALL_2) cells in the logarithmic growth phase were collected, washed twice with pre-chilled PBS. The grinded cells was treated with cell lysis buffer and nucleus was collected by centrifuging at 2000g for 5min. Then, nucleus was treated with nucleus lysis buffer and sonicated to fragment chromatin DNA. The 10% lysis sonicated chromatin was stored and named “input”, and 80% was used in immunoprecipitation reactions with anti-BRG1 antibody（abcam, ab110641）and named “IP”, and 10% was incubated with rabbit IgG (Cell Signaling Technology, #2729) as a negative control and named “IgG”, respectively. The DNA of input and IP was extracted by phenol-chloroform method. The high-throughput DNA sequencing libraries were prepared by using VAHTS Universal DNA Library Prep Kit for Illumina V3（Catalog NO. ND607, Vazyme）. The library products corresponding to 200-500 bps were enriched, quantified and finally sequenced on DNBSEQ-T7 sequencer (MGI Tech Co., Ltd. China) with PE150 model.

Data Processing

a. Raw sequencing data was first filtered by Trimmomatic (version 0.36), low-quality reads were discarded and the reads contaminated with adaptor sequences were trimmed. The clean reads were used for protein binding site analysis. They were mapped to the reference genome hg38 of human using STAR software (version 2.5.3a) with default parameters. The RSeQC (version 2.6) was used for reads distribution analysis.The MACS2 software (Version 2.1.1) was used for peak calling. The bedtools (Version 2.25.0) was used for peaks annotation and peak distribution analysis. The differentially binding peaks were identified by a python script, using fisher test. The Homer (version 4.10) was used for motifs analysis. GO analysis and KEGG enrichment analysis for annotated genes were both implemented by KOBAS software (version: 2.1.1) with a corrected P-value cutoff of 0.05 to judge statistically significant enrichment. Data visualisation performed by IGV software (version: 2.17.0).

For ChIP‒qPCR, immunoprecipitation was incubated using anti-BRG1 or normal rabbit IgG antibody overnight at 4 °C. Then, qRT‒PCR was utilized to quantify the immunoprecipitated DNA, and the data were normalized to the input. The primers used for ChIP‒qPCR are listed in [Table S2](https://www.ncbi.nlm.nih.gov/pmc/articles/PMC9723522/#appsec1).

**Plasmid transfection**

Human PPP2R1A overexpression plasmids containing firefly luciferase (PPP2R1A-firefly_Luciferase), empty plasmids containing firefly luciferase (MCS-firefly_Luciferase) and negative-control plasmids containing Renilla luciferase (TK promoter-Renilla_Luciferase) were purchased from Genechem Co., Ltd. (Shanghai, China). RS4:11/Vector and RS4:11/LV-BRG1 cells were cultured to approximately 80% confluence. PPP2R1A-firefly_Luciferase or MCS-firefly_Luciferase was mixed with TK promoter-Renilla_Luciferase in a 10:1 ratio. RS4:11/Vector and RS4:11/LV-BRG1 cells were transiently transfected with the PPP2R1A-firefly_Luciferase–TK Renilla_Luciferase complex or the MCS-firefly_Luciferase–TK p-Renilla_Luciferase complex using the Lipofectamine™ 3000 Transfection Reagent according to the manufacturer’s instructions (L3000015, Thermo Fisher Scientific, USA). All experiments were performed in triplicate.

**Statistical analysis**

The GraphPad Prism (version 8.0) software (GraphPad Software, Inc., USA) was used for statistical analysis. Data were expressed as the mean ± standard deviation (SD). One-way analysis of variance (ANOVA) was used to compare continuous variables among three or more groups, whereas two-tailed Student’s t-test was used to compare the variables between two groups. The LSD test was used for post hoc analysis when the results of ANOVA were significant. Kaplan–Meier curves were plotted to visualise mouse survival, and the log-rank test was used for analysis. Spearman correlation analysis was used to examine the expression of two proteins. A p-value of <0.05 was considered statistically significant.
