# Supplementary material for: BRG1 promotes progression of B-cell acute lymphoblastic leukemia by disrupting PPP2R1A transcription
Source: Cell Death Dis. 2024 Aug 26;15(8):621. doi: 10.1038/s41419-024-06996-w (PMC11347705; doi:10.1038/s41419-024-06996-w)
Supplement: Supplementary file 2 — Legends for supplementary figures and tables [file 41419_2024_6996_MOESM2_ESM.docx]

**Legends for supplementary figures and tables**

Supplementary Figure 1. **BRG1 is overexpressed in B-ALL patients and is associated with worse outcomes.** (A) Hazard ratios of SMARCA4 to multiple tumors in the TARGET database. (B) Scatter-bar plot of mRNA expression of SMARCA4 in TARGET B-ALL dataset. (C) Scatter-bar plot of mRNA expression of SMARCA2 in TARGET B-ALL dataset. (D) Histogram of ICC staining intensity statistics.

Supplementary Figure 2. **BRG1 inhibition suppresses B-ALL cell proliferation and prolongs survival in vivo.**

RS4:11 cells transduced with ectopic BRG1 (LV-BRG1) were transplanted into NTG mice. Subsequently, mice were treated with the placebo (corn oil) or PFI-3 (10 mg/kg per day, intraperitoneal) for 1 week. The survival of diseased mice was monitored. (A) Proportion of CD45+ cells in human B-ALL cells in the angular vein of mice. (B) Proportion of human CD45+ and CD19+ cells in the BM of mice. (C) Survival of transplanted mice (n = 8 per group). (D) Representative gross illustrations of spleen (up) and liver (below) metastases in mice injected with cells. White arrows indicate neoplasm invasiveness. (E) Histogram of spleen length statistics for each group of mice. (F) H&E staining and immunohistochemical (BRG1 and Ki67 expression) analysis of the liver, spleen and femur in the PFI-3 and placebo groups. The histogram shows the average proportion of BRG1- and Ki67-positive cells (*, *P*＜.05; **, *P*＜.01, ***, *P*＜.001).

Supplementary Figure 3. **BRG1-mediated anti-apoptosis effects rely on the PI3K/AKT signalling pathway.**

RS4:11/LV-BRG1 cells and RS4:11/Vector cells were treated with a vehicle (DMSO) or Ly294002 (50 nM) for 24 hours. (A) Apoptosis in each group was analysed via 7-ADD/annexin V labelling. Apoptosis rate is equal to the sum of the frequencies of the two right quadrants (which correspond to early apoptosis (bottom) and late apotosis/necrosis (upper). (B) Western blotting was used to identify markers of B-ALL cell lines in different drug administration groups. (C) The apoptosis rate for each drug administration group is displayed in the histogram. (D) Histogram demonstrating the relative grey values. All experiments were repeated three times independently. All data are expressed as the mean ± standard error of the mean (*, *P*＜.05; **, *P*＜.01; ***, *P*＜.001; ns, not significant).

Supplementary Figure 4**. Identification of genome-wide DNA binding sites for BRG1 by ChIP-seq.** ChIP-Seq was performed on SUP-B15 cells (ALL_1) and Nalm-6 cells (ALL_2). (A) Statistical plots of raw data processed using the software fastp (version: 0.22.0) for quality control. (B) Clean reads bases quality distribution. (C) Distribution of Reads on Chromosomes. In the ChIP-seq analysis of transcription factors with Input and IP, we took Input as the background and used Macs2 to call peak for IP, and narrow peak mode for peak calling. (D) Peaks Information Sheet. (E) Pie chart of the distribution of Reads across different regions on the reference genome. Signals in the 2-kb region upstream and downstream of the transcription start site (TSS) were counted using the computeMatrix module of DeepTools software. (F) Mean signal distribution over a 2-kb region upstream and downstream of the TSS for each sample.

Supplementary Figure 5**. ChIP-seq plot of the indicated gene in SUP-B15 and Nalm-6 cells.** ChIP-Seq was performed on SUP-B15 cells (ALL_1) and Nalm-6 cells (ALL_2). (A) ChIP-seq profiling showed the ChIP-seq signal for BRG1 at the genomic loci of Myc in ALL_1 (-log10(p-value)=8.00) and ALL_2 (-log10(p-value)=2.80). Histograms of significant differences are shown on the right side. (B) ChIP-seq profiling showed the ChIP-seq signal for BRG1 at the genomic loci of PPP2R1B in ALL_1 and ALL_2 cells. (C) Predicted BRG1 binding to PPP2R1A motifs (*P*=0.01). (D) The correlation between PPP2R1A and Myc was analysed at the protein level using TMT-labeled proteomics results. (E) Histograms show ChIP-seq signals for BRG1 at the promoters of the PPP2R1A gene in ALL_1 (-log10(p-value)=7.13)) and ALL_2 (-log10(p-value)=3.20). (-log10(p-value)＞1.3, *P＜*0.05).

Supplementary Table 1. **Specific characteristics of patients and disease.**

Supplementary Table 2. **Sequences of PCR primers, ChIP-qPCR primers, siRNAs and shRNAs.**

Supplementary Table 3. **List of 114 protein up-regulated and 51 protein down-regulated genes by silencing BRG1.**
