# Supplementary figures and images for: BRG1 promotes progression of B-cell acute lymphoblastic leukemia by disrupting PPP2R1A transcription
Source: Cell Death Dis. 2024 Aug 26;15(8):621. doi: 10.1038/s41419-024-06996-w (PMC11347705; doi:10.1038/s41419-024-06996-w)

Supplementary Figure 1

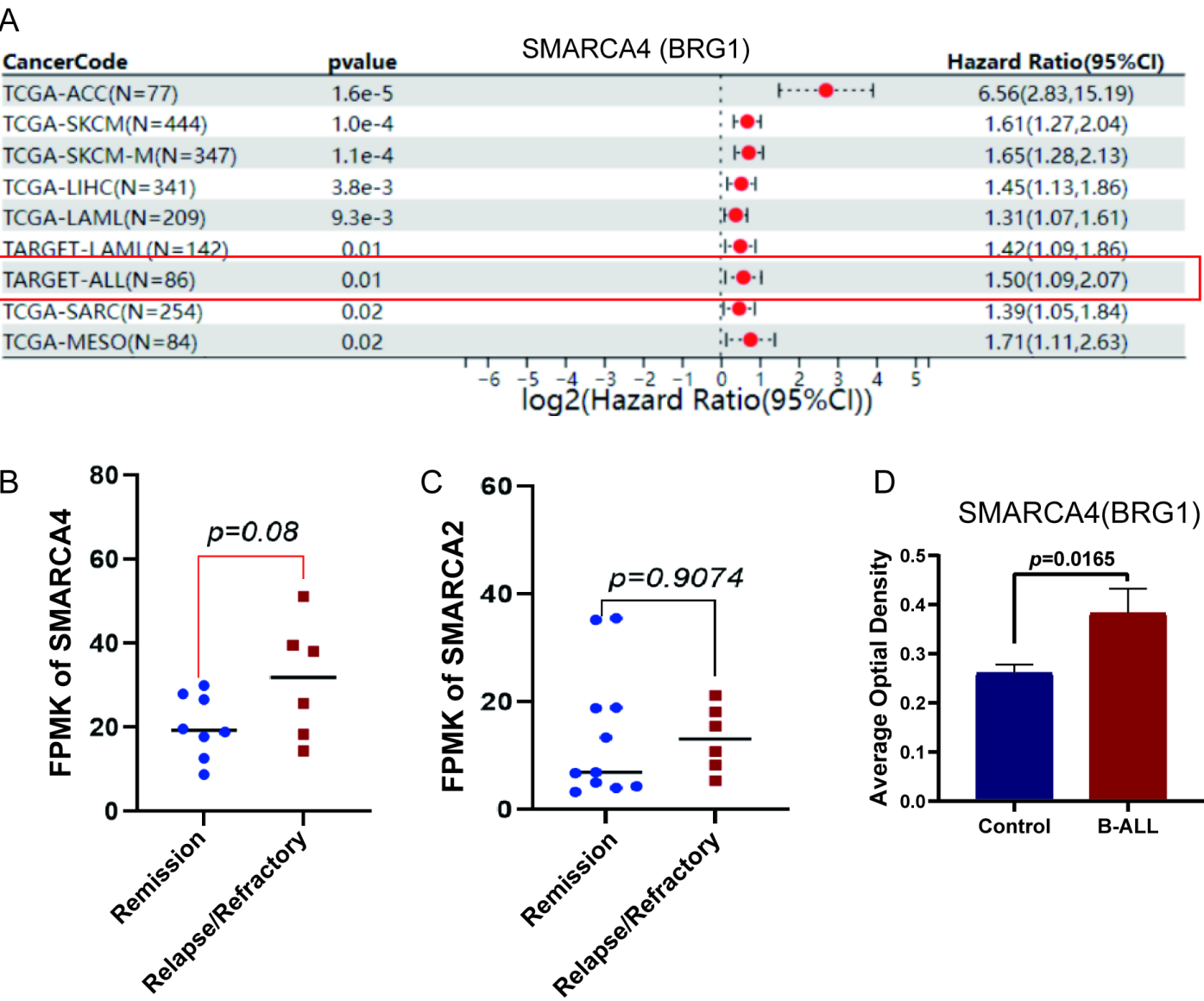

Supplementary Figure 2

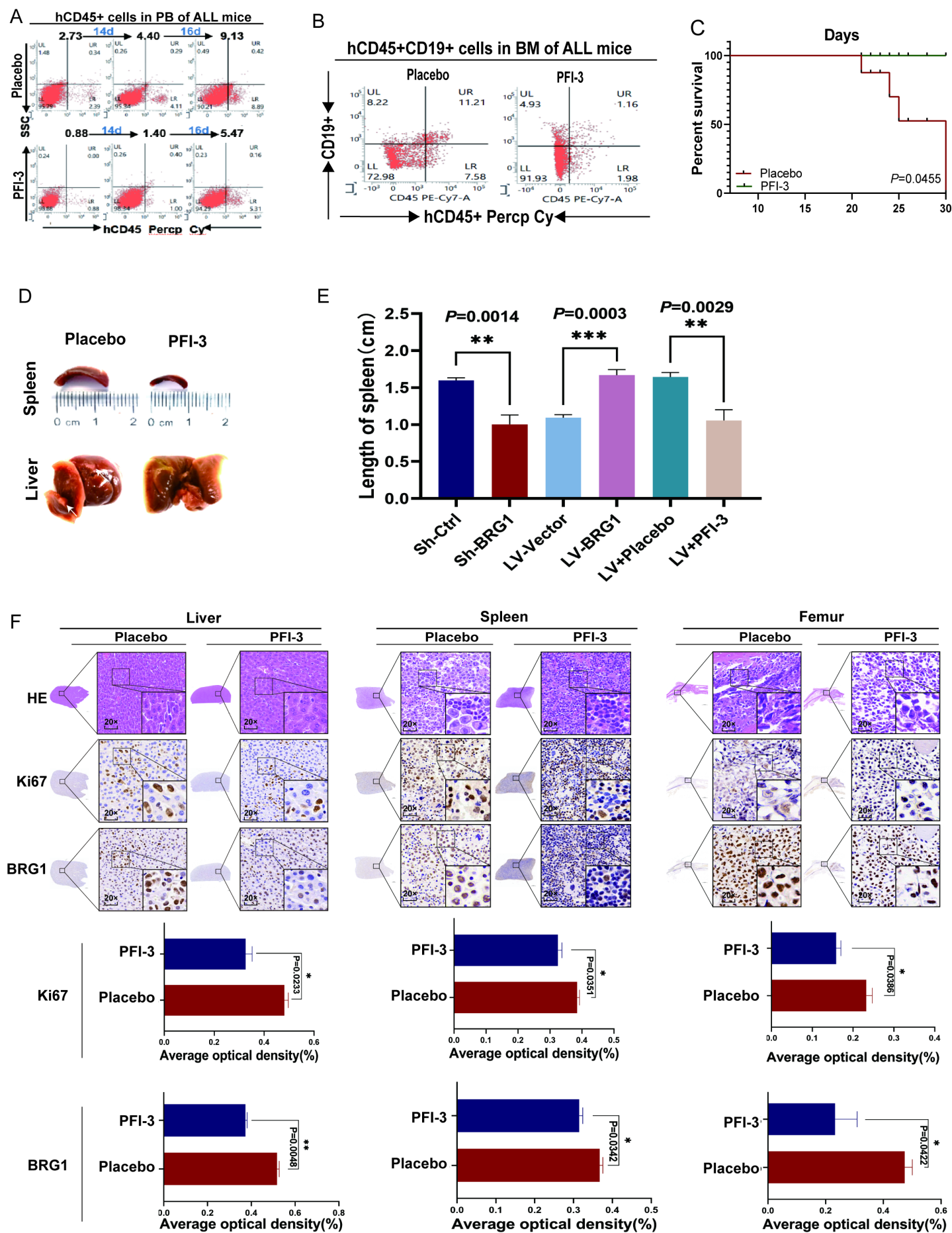

Supplementary Figure 3

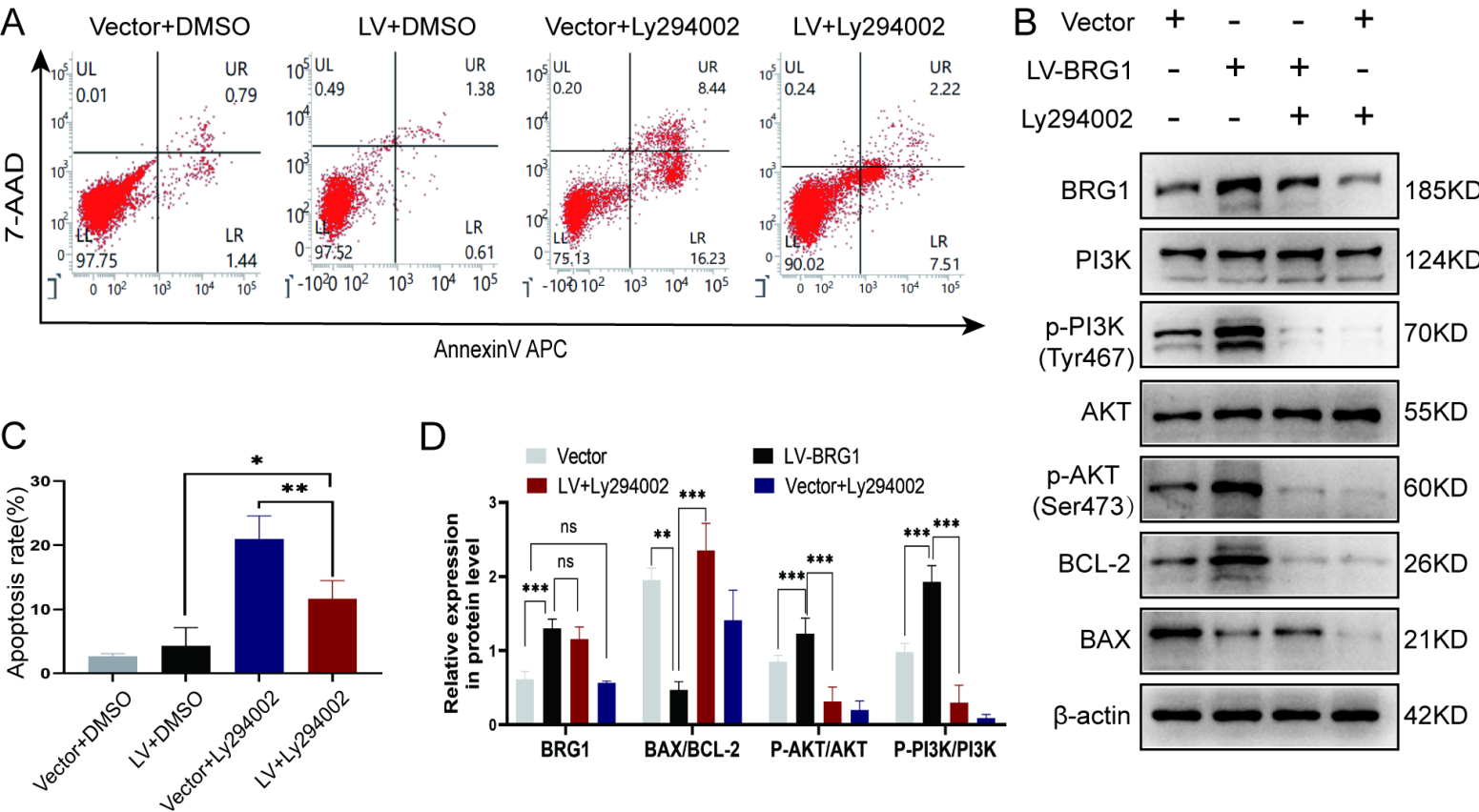

## A

# B

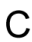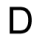F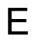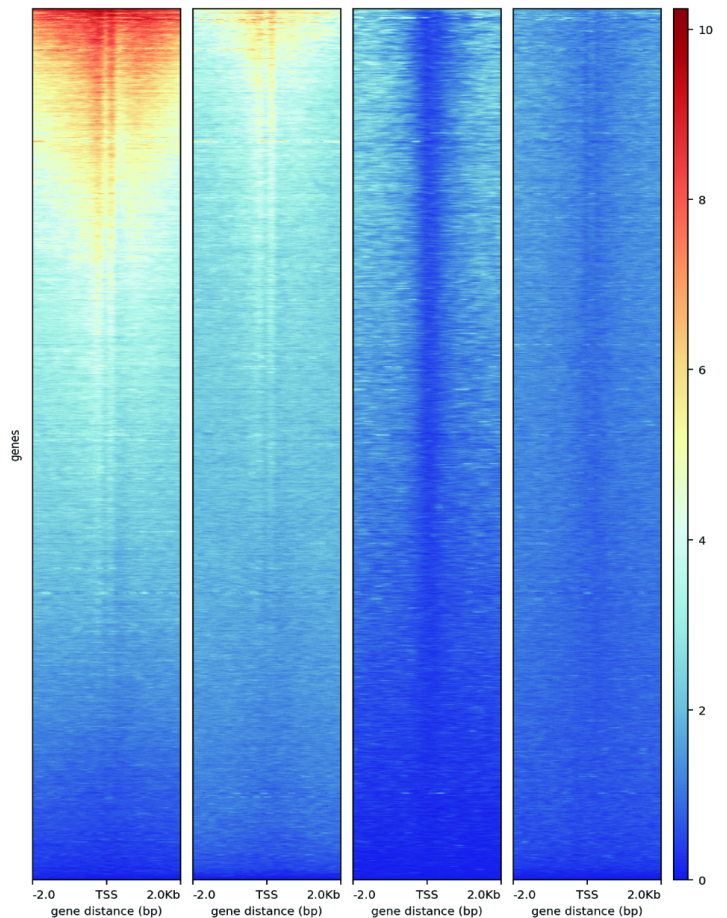

Supplementary Figure 5

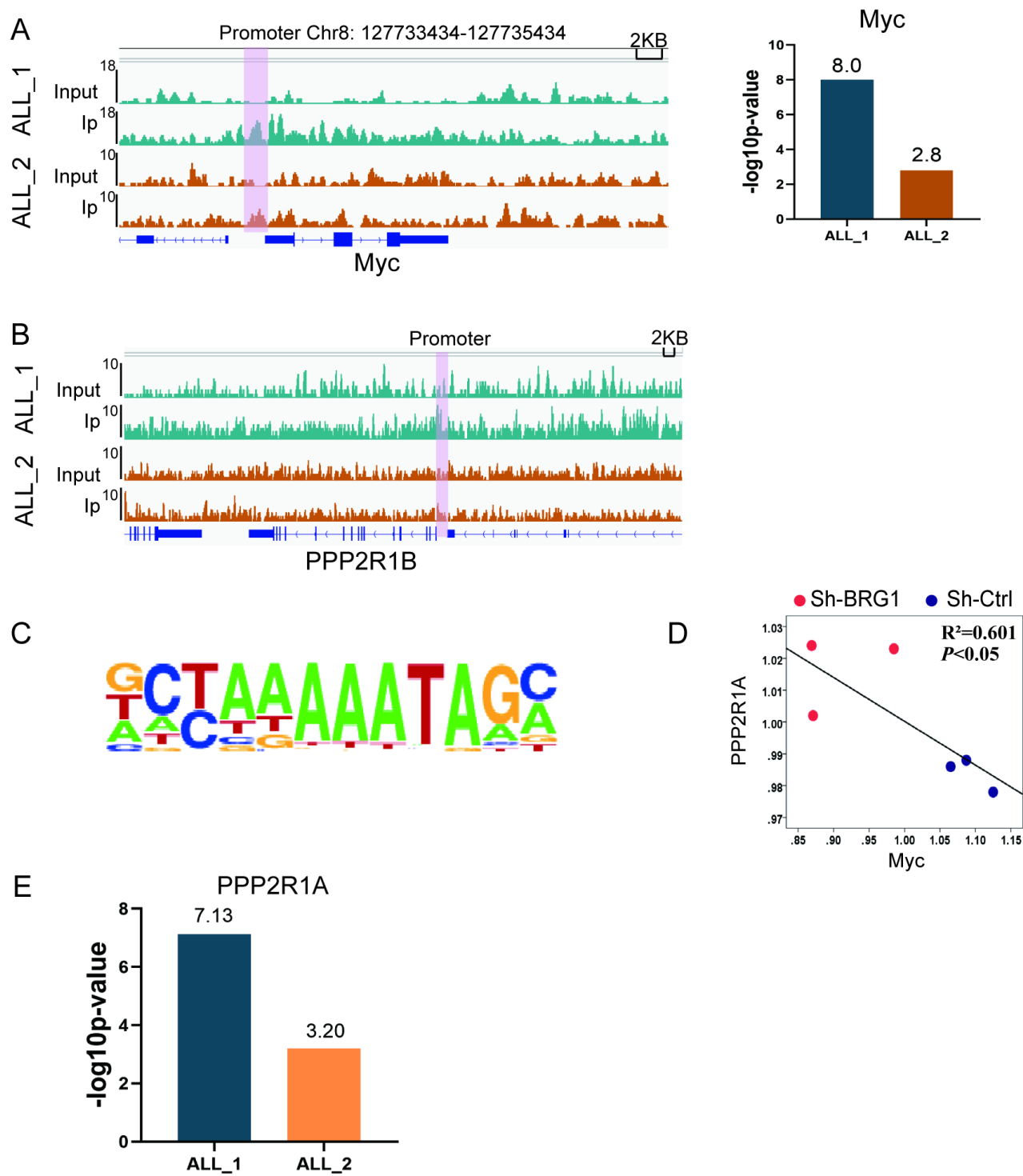

Supplement: Supplementary file 3 — Supplementary Figure 1-5 [file 41419_2024_6996_MOESM3_ESM.pdf]
