# Supplementary material for: BRG1 promotes progression of B-cell acute lymphoblastic leukemia by disrupting PPP2R1A transcription
Source: Cell Death Dis. 2024 Aug 26;15(8):621. doi: 10.1038/s41419-024-06996-w (PMC11347705; doi:10.1038/s41419-024-06996-w)
Supplement: Supplementary file 4 — Supplementary Table 1 [file 41419_2024_6996_MOESM4_ESM.docx]

**Supplementary Table 1. Specific characteristics of patients and disease**

| **ID sample** | **Gender** | **Age**  **(years)** | **Hematological index** | | | **Source** | **Fusion gene** | Blast percent-age in BM（FACS） | Subtype | mutant |
| --- | --- | --- | --- | --- | --- | --- | --- | --- | --- | --- |
|  |  |  | **WBC**  **(G/L)** | **HGB**  **（g/l）** | **PLT**  **(G/L)** |  |  |  |  |  |
| ALL001 | F | 24 | 2.04 | 60 | 56 | BM | BCR-ABL(P190)、HOX11 | 45.82% | Pre-B |  |
| ALL002 | F | 31 | 0.12 | 60 | 16 | BM | BCR-ABL(P210) | 47.62 | COM-B |  |
| ALL003 | F | 58 | 9.47 | 122 | 3 | BM | BCR-ABL(P190) | 73% | COM-B |  |
| ALL004 | M | 18 | 117 | 49 | 34 | BM |  | 89.28 | COM-B | FLT3-D835 |
| ALL005 | F | 47 | 9.19 | 89 | 6 | BM | BCR-ABL(P210)、HOX11、WT1 | 50.52 | COM-B |  |
| ALL006 | F | 43 | 180.46 | 75 | 49 | BM | BCR-ABL(P190) | 75.6 | Pre-B |  |
| ALL007 | M | 63 | 0.63 | 67 | 96 | BM |  | 76.52 | Pre-B |  |
| ALL008 | M | 18 | 1.82 | 79 | 11 | BM | E2A/PBX1 | 80 | COM-B |  |
| ALL009 | M | 42 | 115.64 | 59 | 34 | BM |  | 84.22 | Pro-B-ALL |  |
| ALL010 | M | 53 | 24.04 | 76 | 44 | BM | WT1 | 87.88 | Pre-B-ALL |  |
| ALL011 | M | 38 | 7.98 | 62 | 116 | BM |  | 62.59 | B-ALL |  |
| ALL012 | M | 43 | 21.95 | 136 | 19 | BM | BCR-ABL(P210) | 52.58 | COM-B |  |
| ALL013 | M | 46 | 4.31 | 93 | 13 | BM |  | 56.23 | Pre-B-ALL |  |
| ALL014 | M | 19 | 28.69 | 68 | 3 | BM |  | 77.76 | COM-B |  |
| ALL015 | F | 45 | 261.6 | 131 | 25 | BM | BCR-ABL(P190) | 81.58 | COM-B |  |
| ALL016 | F | 24 | 2.04 | 60 | 56 | BM | BCR-ABL(P190) | 45.82 | Pre-B-ALL |  |
| ALL017 | M | 46 | 4.31 | 93 | 13 | BM |  | 56.23 | Pre-B-ALL |  |
| ALL018 | F | 31 | 5.93 | 51 | 99 | BM |  | 73.11 | COM-B |  |
| ALL019 | M | 50 | 1.98 | 38 | 22 | BM |  | 77.76 | COM-B |  |
| ALL020 | M | 22 | 31.52 | 140 | 15 | BM |  | 88.29 | COM-B |  |
| ALL021 | M | 21 | 1.67 | 54 | 146 | BM | BCR-ABL(P190) | 88.27 | Pre-B-ALL |  |
| ALL022 | F | 46 | 23.09 | 62 | 38 | BM | BCR-ABL(P190) | 75.93 | Pre-B-ALL | ASXL1-13exonA |
| ALL023 | M | 46 | 4.13 | 93 | 13 | BM |  | 56.23 | Pre-B-ALL |  |
| ALL024 | F | 40 | 4.57 | 101 | 81 | BM | BCR-ABL(P190) | 66.23 | COM-B |  |
| ALL025 | F | 62 | 3.92 | 65 | 22 | BM |  | 72.23 | COM-B | TP53 |
| ALL026 | F | 18 | 29.43 | 112 | 25 | BM |  | 78.92 | COM-B | FLT3-ITD |
| ALL027 | M | 19 | 3.1 | 55 | 83 | BM |  | 41.4 | COM-B | FLT3-ITD |
| ALL028 | F | 21 | 3.47 | 58 | 89 | BM | EVT6-RUNX1 | 76.55 | COM-B |  |
| ALL029 | M | 20 | 0.94 | 63 | 63 | BM |  | 94.78 | COM-B |  |
| ALL030 | M | 43 | 19.34 | 116 | 19 | BM |  | 73.61 | COM-B |  |
| ALL031 | M | 20 | 36.35 | 59 | 36 | BM |  | 74.68 | COM-B |  |
