# Supplementary material for: BRG1 promotes progression of B-cell acute lymphoblastic leukemia by disrupting PPP2R1A transcription
Source: Cell Death Dis. 2024 Aug 26;15(8):621. doi: 10.1038/s41419-024-06996-w (PMC11347705; doi:10.1038/s41419-024-06996-w)
Supplement: Supplementary file 5 — Supplementary Table 2 [file 41419_2024_6996_MOESM5_ESM.docx]

Supplemental Table 2

| Sequences of ShRNAs | | | |
| --- | --- | --- | --- |
| Sh-SMARCA4#1 | | CAGGTGCTCAACACGCACTAT | |
| Sh-SMARCA4#2 | | CCGCGCTACAACCAGATGAAA | |
| Sh-SMARCA4#3 | | TACCGAGCCTCGGGTAAATTT | |
|  | |  | |
| Sequences of primers for siRNAs（5’ to 3’） | | | |
| siRNA-c-Myc#1 | | CAAGGUAGUUAUCCUUAAATT | |
|  |  | UUUAAGGAUAACUACCUUGTT | |
| siRNA-c-Myc#2 | | UGAAAGAUUUAGCCAUAAUTT | |
|  |  | AUUAUGGCUAAAUCUUUCATT | |
| siRNA-c-Myc#3 | | ACGGAACUCUUGUGCGUAATT | |
|  |  | UUACGCACAAGAGUUCCGUTT | |
|  | |  | |
| Sequences of primers for PCR | | | |
| SMARCA4 | Forward primer | | GCCCATTGATGCGTTTGT |
|  | Reverse primer | | GGTTTGGAGTGGCTGGTG |
| c-Myc | Forward primer | | CCTGGTGCTCCATGAGGAGAC |
|  | Reverse primer | | CAGACTCTGACCTTTTGCCAGG |
| β-actin | Forward primer | | CCTGGCACCCAGCACAAT |
|  | Reverse primer | | GGGCCGGACTCGTCATAC |
| PPP2R1A | Forward primer | | AAAGGGACGGAGCCAAGATG |
|  | Reverse primer | | GGGTAGTGAAGGTTCCCAGC |
|  |  | |  |
|  |  | |  |
| Sequences of primers for ChIP-qPCR | | | |
| PPP2R1A | Forward primer | | CCAGCTAGTGACATGGGTGC |
|  | Reverse primer | | TAACACGCTTCCTCTGTGCC |
|  |  | |  |
|  |  | |  |
