# Supplementary material for: BRG1 promotes progression of B-cell acute lymphoblastic leukemia by disrupting PPP2R1A transcription
Source: Cell Death Dis. 2024 Aug 26;15(8):621. doi: 10.1038/s41419-024-06996-w (PMC11347705; doi:10.1038/s41419-024-06996-w)
Supplement: Supplementary file 6 — Supplementary Table 3 [file 41419_2024_6996_MOESM6_ESM.docx]

**Supplemental Table 3**

| **Gene name** | **Sh_BRG1/Sh_ctrl Ratio** | **Sh_BRG1/Sh_ctrl P value** | **Regulated Type** | **MW [kDa]** |
| --- | --- | --- | --- | --- |
| MAT1A | 1.223 | 0.009345994 | Up | 43.6 |
| P4HA2 | 1.217 | 0.02158139 | Up | 60.9 |
| RGN | 1.513 | 0.000478922 | Up | 33.2 |
| GAS7 | 1.225 | 0.000528747 | Up | 54.3 |
| H2AX | 1.334 | 0.000435861 | Up | 15.1 |
| SCD5 | 1.484 | 0.005231479 | Up | 37.6 |
| STRN4 | 1.836 | 0.000535984 | Up | 80.5 |
| RPS28 | 1.21 | 0.000315614 | Up | 7.8 |
| RPL18 | 1.22 | 0.006162908 | Up | 21.6 |
| CHCHD6 | 1.224 | 0.006609531 | Up | 26.4 |
| PRC1 | 1.255 | 0.000674662 | Up | 71.6 |
| PTTG1IP | 1.202 | 0.00252615 | Up | 20.3 |
| SMARCA2 | 1.232 | 4.75955E-06 | Up | 181.2 |
| TRIR | 1.239 | 0.001133626 | Up | 18.4 |
| VKORC1 | 1.406 | 0.001683622 | Up | 18.2 |
| JARID2 | 1.351 | 0.004598623 | Up | 138.6 |
| TYW1 | 1.22 | 0.008689507 | Up | 83.6 |
| SLC25A25 | 1.35 | 0.000843402 | Up | 52.6 |
| ACP6 | 1.211 | 0.002879772 | Up | 48.9 |
| SLC29A1 | 1.208 | 0.003719802 | Up | 50.2 |
| PLD4 | 1.294 | 0.003993744 | Up | 55.6 |
| ITGB2 | 1.248 | 0.000388231 | Up | 84.7 |
| METTL7A | 1.209 | 0.010143515 | Up | 28.3 |
| LIPA | 1.221 | 0.004751286 | Up | 45.4 |
| TP53I11 | 1.473 | 0.017627078 | Up | 21 |
| MOB3A | 1.288 | 0.01838817 | Up | 25.4 |
| MYO1C | 1.264 | 0.038826422 | Up | 121.6 |
| ADSS1 | 1.325 | 0.018911054 | Up | 50.2 |
| ARHGAP12 | 1.205 | 0.029007858 | Up | 96.2 |
| HMGB2 | 1.314 | 0.004306144 | Up | 24 |
| MUL1 | 1.33 | 0.000907559 | Up | 39.8 |
| H1-4 | 1.253 | 0.011584545 | Up | 21.9 |
| NHLRC3 | 1.36 | 0.014758154 | Up | 38.3 |
| UBOX5 | 1.201 | 0.033479607 | Up | 58.9 |
| OGFOD2 | 1.22 | 0.011671695 | Up | 39 |
| TXN | 1.229 | 0.00018483 | Up | 11.7 |
| ZNF267 | 1.233 | 0.036320024 | Up | 87.3 |
| WASHC3 | 1.222 | 0.022539321 | Up | 21.2 |
| PYCR2 | 1.22 | 0.00686658 | Up | 33.6 |
| RRBP1 | 1.203 | 4.56983E-05 | Up | 152.4 |
| PTBP2 | 1.364 | 0.000827 | Up | 57.5 |
| SLC66A2 | 1.42 | 0.000544764 | Up | 30.5 |
| SLC41A3 | 1.263 | 0.013820903 | Up | 54.7 |
| CD79A | 1.27 | 0.00156653 | Up | 25 |
| H1-5 | 1.208 | 0.00111053 | Up | 22.6 |
| HMGN1 | 1.318 | 0.000733845 | Up | 10.7 |
| GPATCH2L | 1.534 | 0.009302605 | Up | 54.2 |
| CD59 | 1.251 | 0.006489885 | Up | 14.2 |
| AURKA | 1.205 | 0.017262147 | Up | 45.8 |
| DNMT3B | 1.345 | 0.021720401 | Up | 95.7 |
| ARL2BP | 1.283 | 0.000109531 | Up | 18.8 |
| BST2 | 1.235 | 0.011588221 | Up | 19.8 |
| CCDC175 | 1.297 | 0.00474494 | Up | 93.6 |
| C18orf21 | 1.25 | 0.000987201 | Up | 24.8 |
| CHMP2B | 1.204 | 0.001535406 | Up | 23.9 |
| CAMLG | 1.27 | 0.000554953 | Up | 32.9 |
| CTTNBP2NL | 1.539 | 0.000348712 | Up | 70.1 |
| ASF1A | 1.232 | 0.041097769 | Up | 23 |
| BAHCC1 | 1.212 | 0.002096501 | Up | 279.8 |
| ABI2 | 1.279 | 0.044600279 | Up | 55.6 |
| DDIT4 | 1.505 | 0.034813202 | Up | 25.4 |
| CD79B | 1.541 | 0.000279934 | Up | 26 |
| CHEK1 | 1.202 | 3.06788E-05 | Up | 54.4 |
| CENPE | 1.233 | 0.001172672 | Up | 316.2 |
| ADA2 | 1.378 | 0.000716412 | Up | 58.9 |
| PPCDC | 1.229 | 0.014273576 | Up | 22.4 |
| CD81 | 1.275 | 0.000615447 | Up | 25.8 |
| DNAL4 | 1.309 | 0.003383957 | Up | 12 |
| CXCR4 | 1.241 | 0.006652128 | Up | 39.7 |
| C1orf112 | 1.296 | 0.001369701 | Up | 96.5 |
| CENPM | 1.219 | 0.025282767 | Up | 19.7 |
| DSC2 | 1.699 | 1.25105E-06 | Up | 99.9 |
| CHEK2 | 1.266 | 5.06297E-05 | Up | 60.9 |
| HMGB3 | 1.227 | 0.000835955 | Up | 23 |
| TM7SF2 | 1.268 | 0.044696735 | Up | 46.4 |
| DDX11 | 1.389 | 0.002025376 | Up | 108.2 |
| CLPTM1 | 1.239 | 0.007329788 | Up | 76 |
| D2HGDH | 1.227 | 0.007513747 | Up | 56.4 |
| FTH1 | 1.239 | 0.003040636 | Up | 21.2 |
| COTL1 | 1.286 | 1.58199E-05 | Up | 15.9 |
| CHCHD5 | 1.292 | 0.006117583 | Up | 12.4 |
| CPTP | 1.233 | 0.039685268 | Up | 24.4 |
| CHTOP | 1.251 | 0.003392069 | Up | 26.4 |
| FAM83D | 1.234 | 0.008906253 | Up | 64.4 |
| BNIP3 | 1.969 | 0.002637043 | Up | 27.8 |
| GSN | 1.239 | 0.000622456 | Up | 85.6 |
| HMMR | 1.233 | 0.000151186 | Up | 84 |
| SLC37A4 | 1.201 | 0.002971644 | Up | 46.3 |
| GRN | 1.318 | 0.017182211 | Up | 63.5 |
| FN1 | 1.25 | 0.012681635 | Up | 272.2 |
| FTL | 1.329 | 0.003328122 | Up | 20 |
| KIF5A | 1.25 | 0.000378813 | Up | 117.3 |
| HCFC2 | 1.277 | 0.001618181 | Up | 86.7 |
| MMADHC | 1.465 | 0.002719162 | Up | 32.9 |
| NCK2 | 1.366 | 0.00506 | Up | 42.9 |
| MREG | 1.242 | 0.047090407 | Up | 24.9 |
| MYH1 | 1.31 | 0.006301832 | Up | 223 |
| TRIP13 | 1.229 | 0.002751048 | Up | 48.5 |
| SLC25A22 | 1.228 | 0.010794588 | Up | 34.4 |
| ENO2 | 1.205 | 0.003462288 | Up | 47.2 |
| NT5DC2 | 1.223 | 0.001028382 | Up | 60.7 |
| F13A1 | 1.516 | 0.000475798 | Up | 83.2 |
| THBS1 | 1.304 | 0.000367464 | Up | 129.3 |
| USE1 | 1.265 | 0.005478074 | Up | 29.4 |
| DNAJC17 | 1.231 | 0.005989421 | Up | 34.7 |
| ZNF652 | 1.211 | 0.02184242 | Up | 69.7 |
| PSRC1 | 1.243 | 0.008441212 | Up | 38.8 |
| PPIL1 | 1.249 | 0.00164463 | Up | 18.2 |
| QPRT | 1.209 | 7.7333E-05 | Up | 30.8 |
| PLBD2 | 1.208 | 0.001543199 | Up | 65.4 |
| TYSND1 | 1.221 | 0.017138988 | Up | 59.3 |
| SYVN1 | 1.217 | 0.000360218 | Up | 67.6 |
| ZNF813 | 1.219 | 0.008762283 | Up | 71.7 |
| ARHGDIB | 1.262 | 0.00016623 | Up | 23 |
| POMP | 0.821 | 0.002874811 | Down | 15.8 |
| RNF114 | 0.815 | 0.000480855 | Down | 25.7 |
| SEPTIN1 | 0.83 | 0.000748213 | Down | 42.4 |
| TKT | 0.819 | 0.000396854 | Down | 67.8 |
| SLC43A1 | 0.789 | 0.022897345 | Down | 61.4 |
| PLEKHO1 | 0.704 | 0.001959559 | Down | 46.2 |
| CROT | 0.829 | 0.013480639 | Down | 70.1 |
| SMARCA4 | 0.67 | 3.26097E-06 | Down | 184.5 |
| RAB12 | 0.807 | 0.002606033 | Down | 27.2 |
| SOAT1 | 0.795 | 0.012730453 | Down | 64.7 |
| NAPG | 0.823 | 5.30727E-05 | Down | 34.7 |
| PBX1 | 0.751 | 0.003957334 | Down | 46.6 |
| ZBTB33 | 0.804 | 0.000148159 | Down | 74.4 |
| NDUFA4 | 0.817 | 0.009338175 | Down | 9.4 |
| MYC | 0.832 | 0.013799261 | Down | 48.8 |
| RAB18 | 0.788 | 0.000222155 | Down | 23 |
| DNTT | 0.776 | 0.000266506 | Down | 58.5 |
| TMA16 | 0.778 | 0.000103779 | Down | 23.8 |
| PCK2 | 0.801 | 0.000246417 | Down | 70.7 |
| UFC1 | 0.803 | 0.000500859 | Down | 19.4 |
| SELENOH | 0.831 | 0.000833393 | Down | 13.4 |
| TASL | 0.796 | 0.011864975 | Down | 33.9 |
| GOPC | 0.798 | 8.25239E-05 | Down | 50.5 |
| TMEM30A | 0.742 | 0.000565186 | Down | 40.7 |
| AFG1L | 0.826 | 0.026143214 | Down | 54.8 |
| APOBEC3C | 0.698 | 0.00232627 | Down | 22.8 |
| C1GALT1 | 0.817 | 0.010973274 | Down | 42.2 |
| CDK6 | 0.794 | 0.000150962 | Down | 36.9 |
| ABHD14B | 0.806 | 0.000703738 | Down | 22.3 |
| CPNE1 | 0.811 | 0.000133506 | Down | 59 |
| ACBD7 | 0.814 | 0.001135191 | Down | 9.8 |
| CHURC1 | 0.821 | 0.000225974 | Down | 16.1 |
| BAG2 | 0.777 | 0.000342265 | Down | 23.8 |
| AKR1A1 | 0.795 | 2.41049E-05 | Down | 36.6 |
| JAK3 | 0.82 | 0.000726076 | Down | 125 |
| IDH1 | 0.737 | 2.43978E-05 | Down | 46.6 |
| GLT8D1 | 0.826 | 0.000679163 | Down | 41.9 |
| GNPTAB | 0.833 | 0.042797223 | Down | 143.5 |
| YWHAH | 0.773 | 4.2977E-05 | Down | 28.2 |
| TSFM | 0.777 | 0.000511008 | Down | 35.4 |
| CS | 0.821 | 0.000115723 | Down | 51.7 |
| LYN | 0.826 | 0.000667275 | Down | 58.5 |
| LMAN2 | 0.779 | 0.003210119 | Down | 40.2 |
| EEF2K | 0.713 | 0.005090841 | Down | 82.1 |
| IKZF5 | 0.764 | 0.00555721 | Down | 46.5 |
| LPXN | 0.75 | 0.007775741 | Down | 43.3 |
| MT1X | 0.662 | 0.040852962 | Down | 6.1 |
| NFKBIL1 | 0.741 | 0.045351218 | Down | 43.2 |
| TTC13 | 0.789 | 0.020152652 | Down | 96.8 |
| UBE2Q2 | 0.82 | 0.004031796 | Down | 42.8 |
| MT2A | 0.508 | 0.006258372 | Down | 6 |
