# Supplementary material for: BRG1 promotes progression of B-cell acute lymphoblastic leukemia by disrupting PPP2R1A transcription
Source: Cell Death Dis. 2024 Aug 26;15(8):621. doi: 10.1038/s41419-024-06996-w (PMC11347705; doi:10.1038/s41419-024-06996-w)

Original western blots

SourceDataF1F

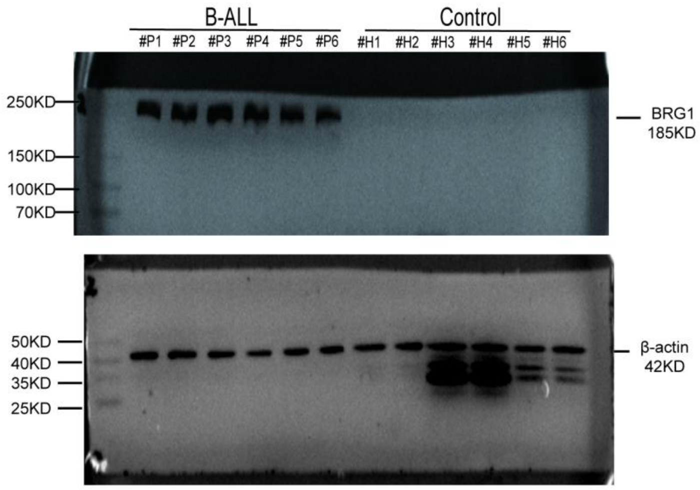

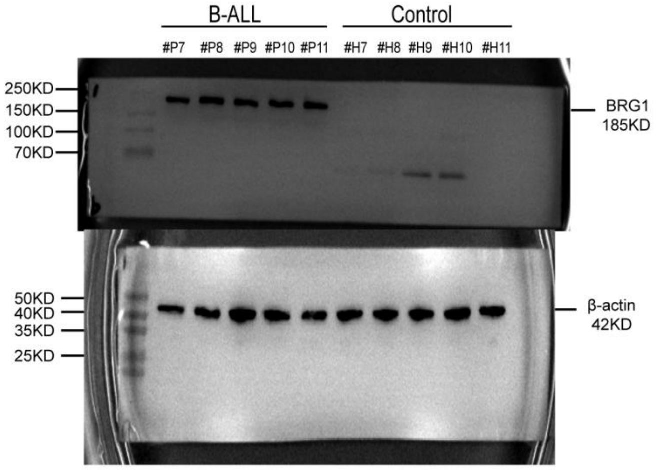

SourceDataF2A

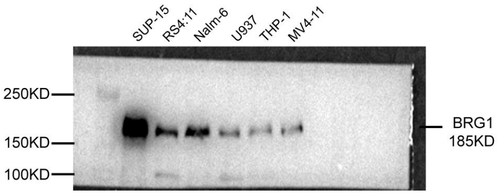

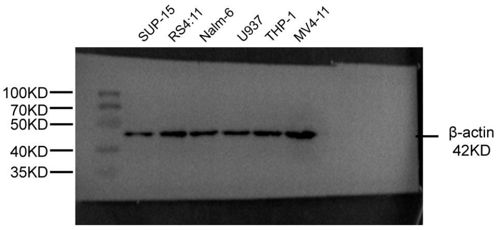

SourceDataF2B

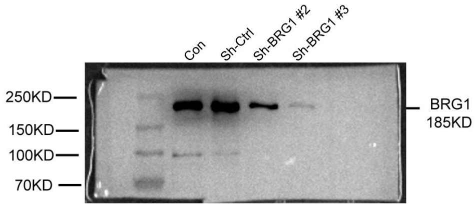

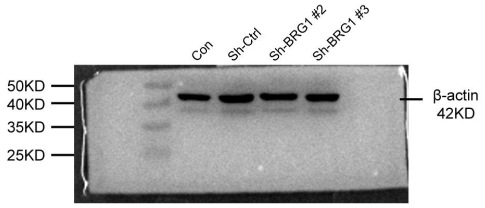

SourceDataF2C

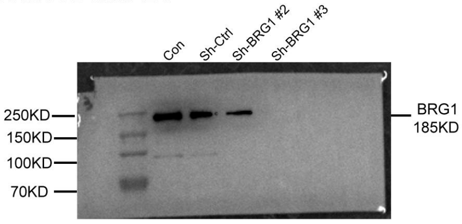

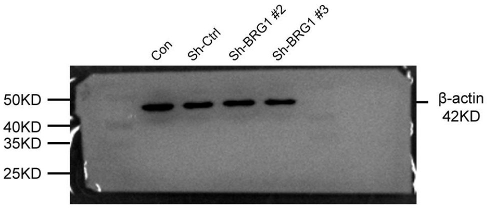

SourceDataF2D

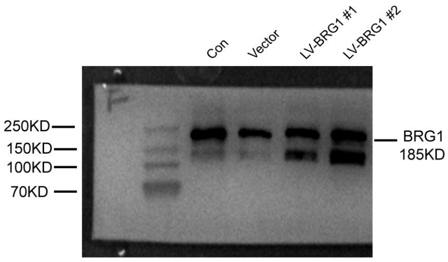

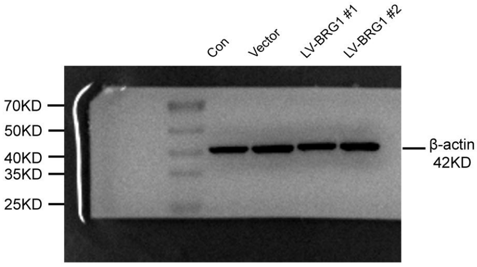

SourceDataF3F

SUP-B15

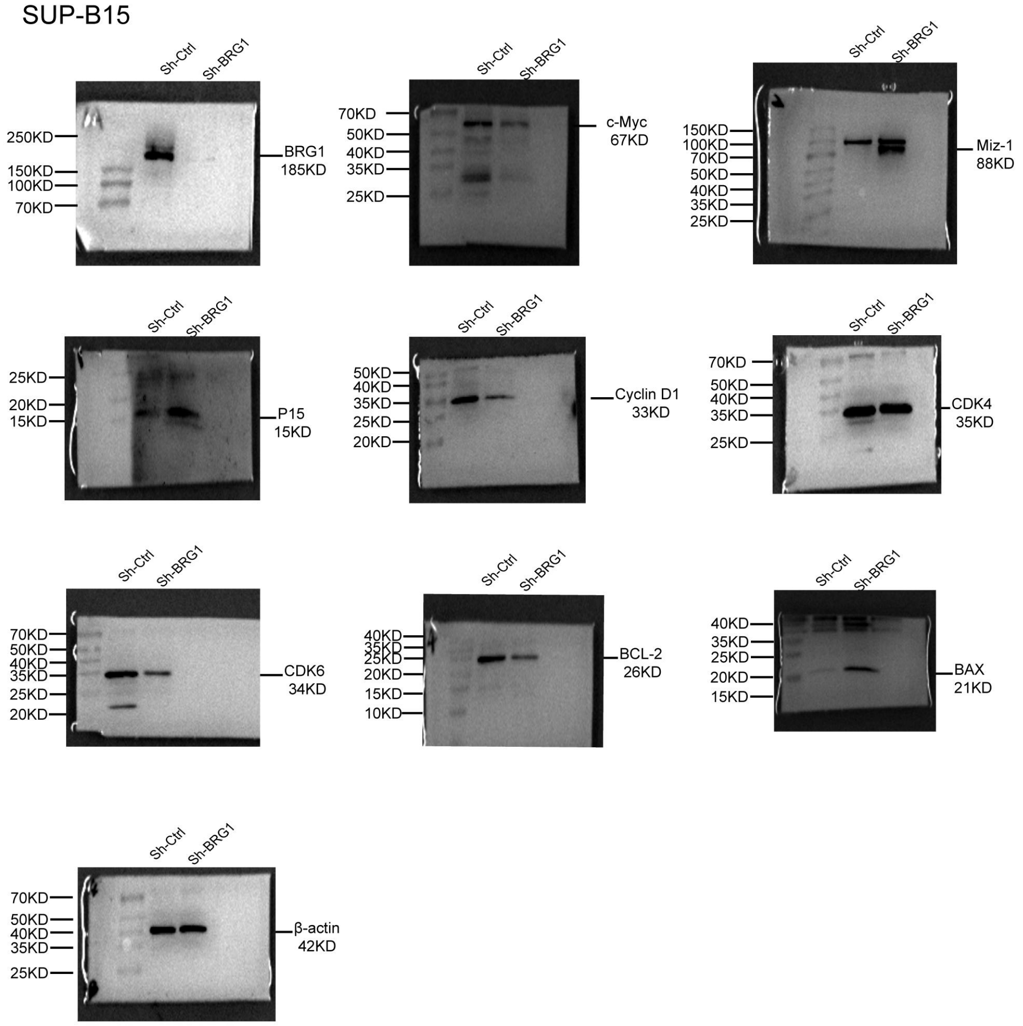

SourceDataF3F

Nalm-6

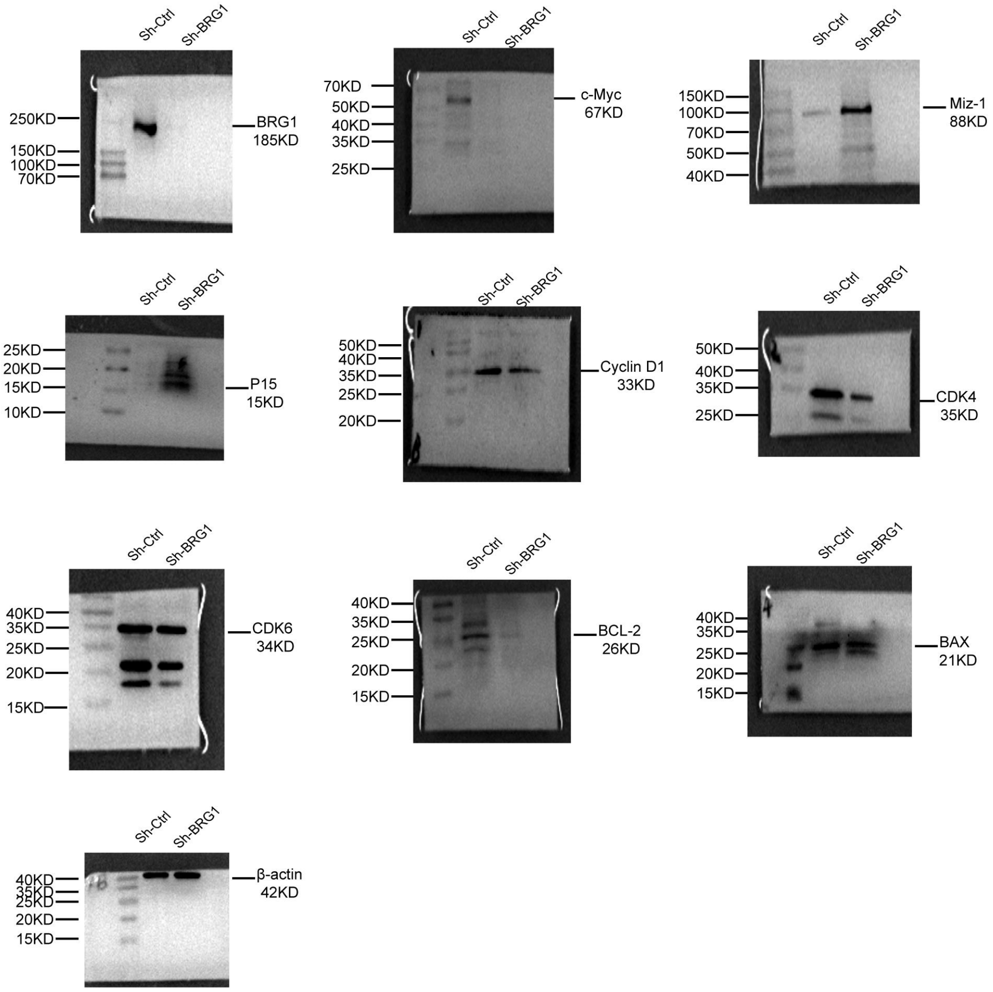

SourceDataF3F

RS 4:11

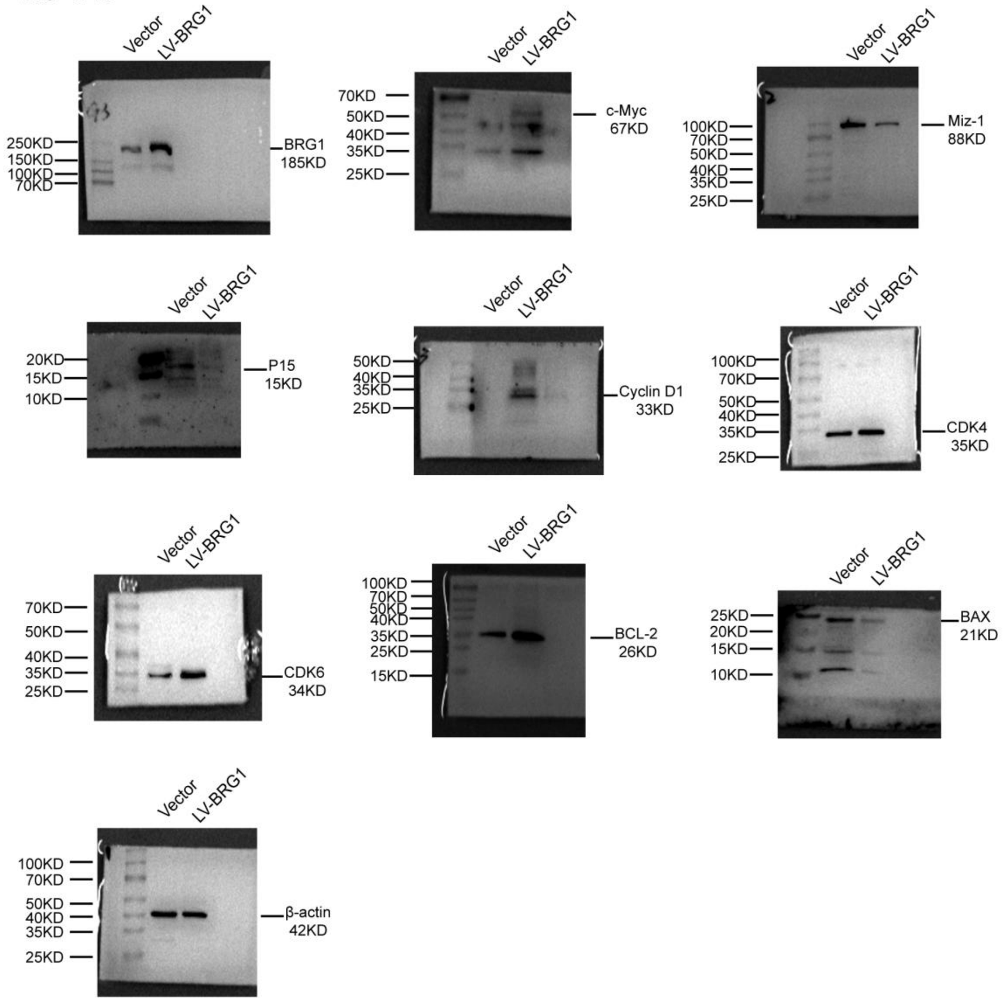

SourceDataF5C

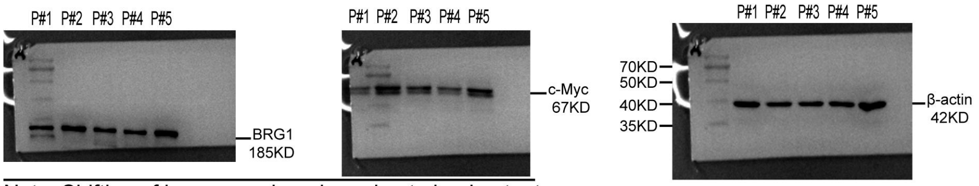

Note: Shifting of images and markers due to inadvertent handling during image acquisition.

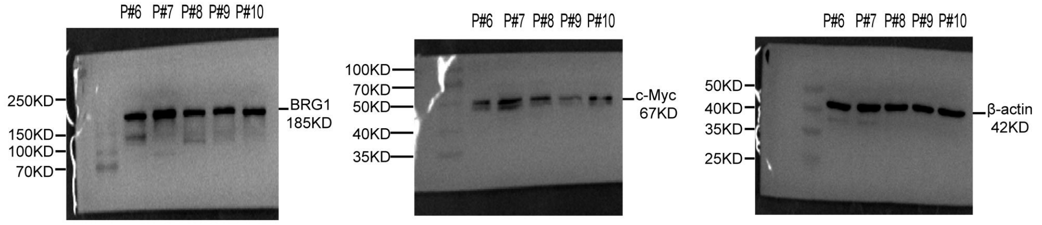

SourceDataF5E

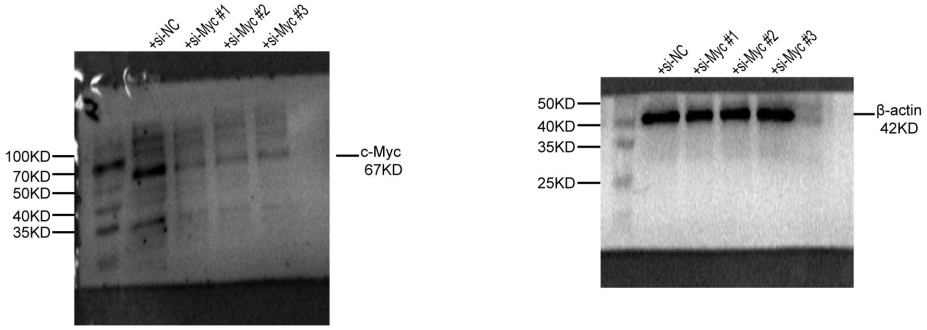

SourceDataF5K

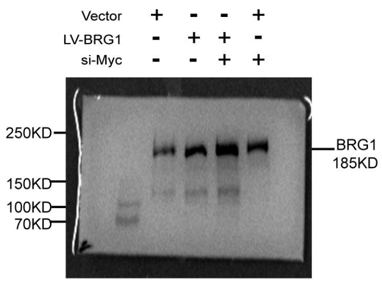

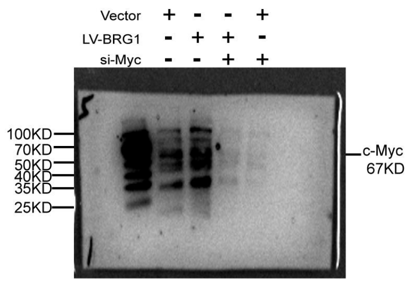

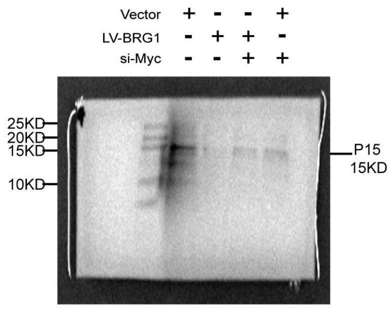

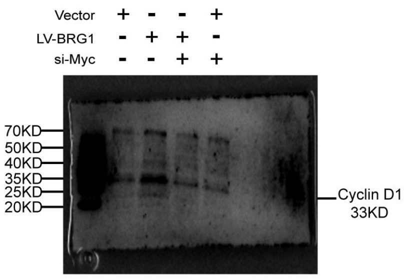

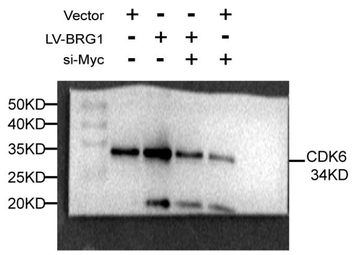

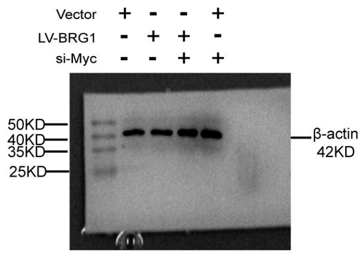

SourceDaraF6E-1

SUP-B15

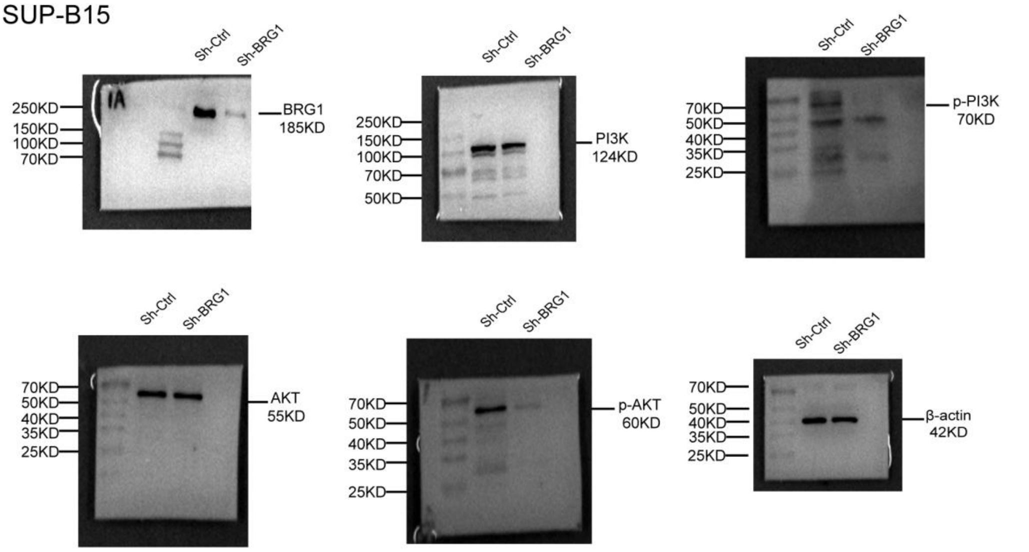

Nalm-6

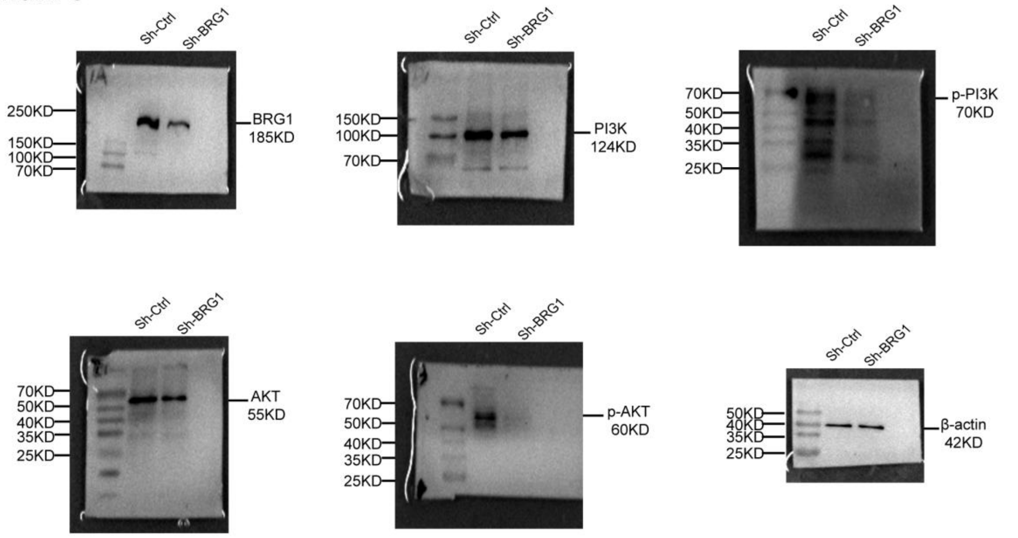

SourceDaraF6E-2

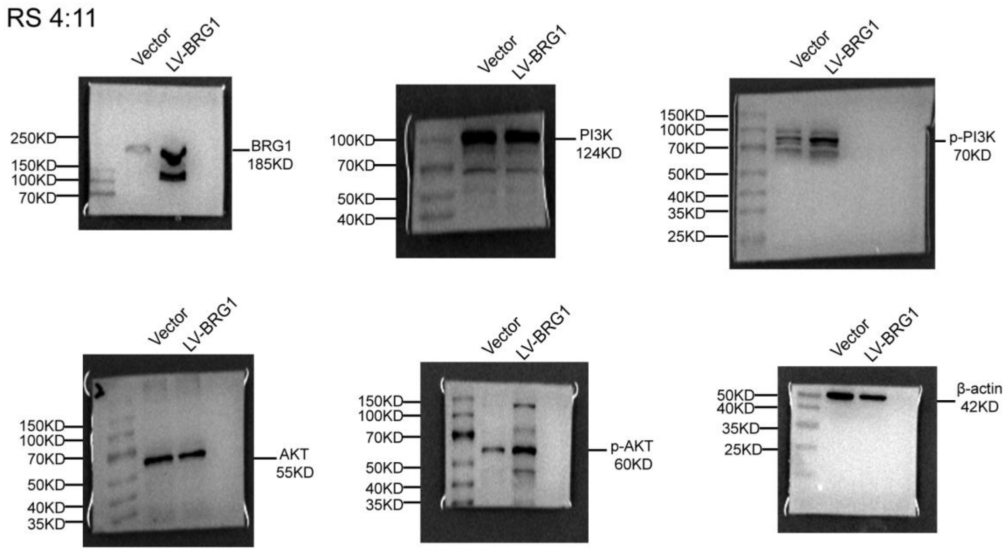

SourceDataF7F

SUP-B15

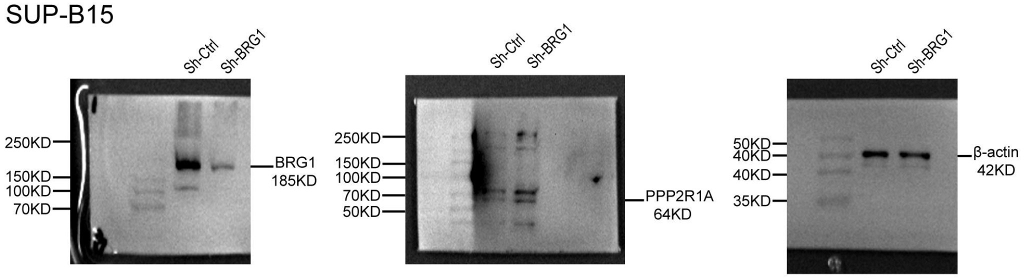

Nalm-6

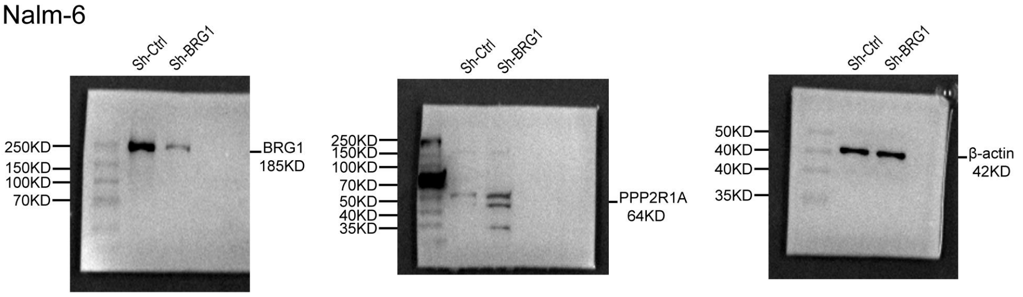

RS4:11

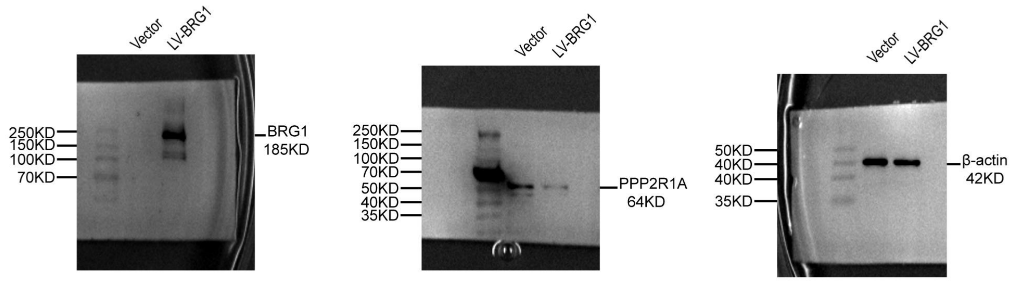

SourceData SupplementalF3B

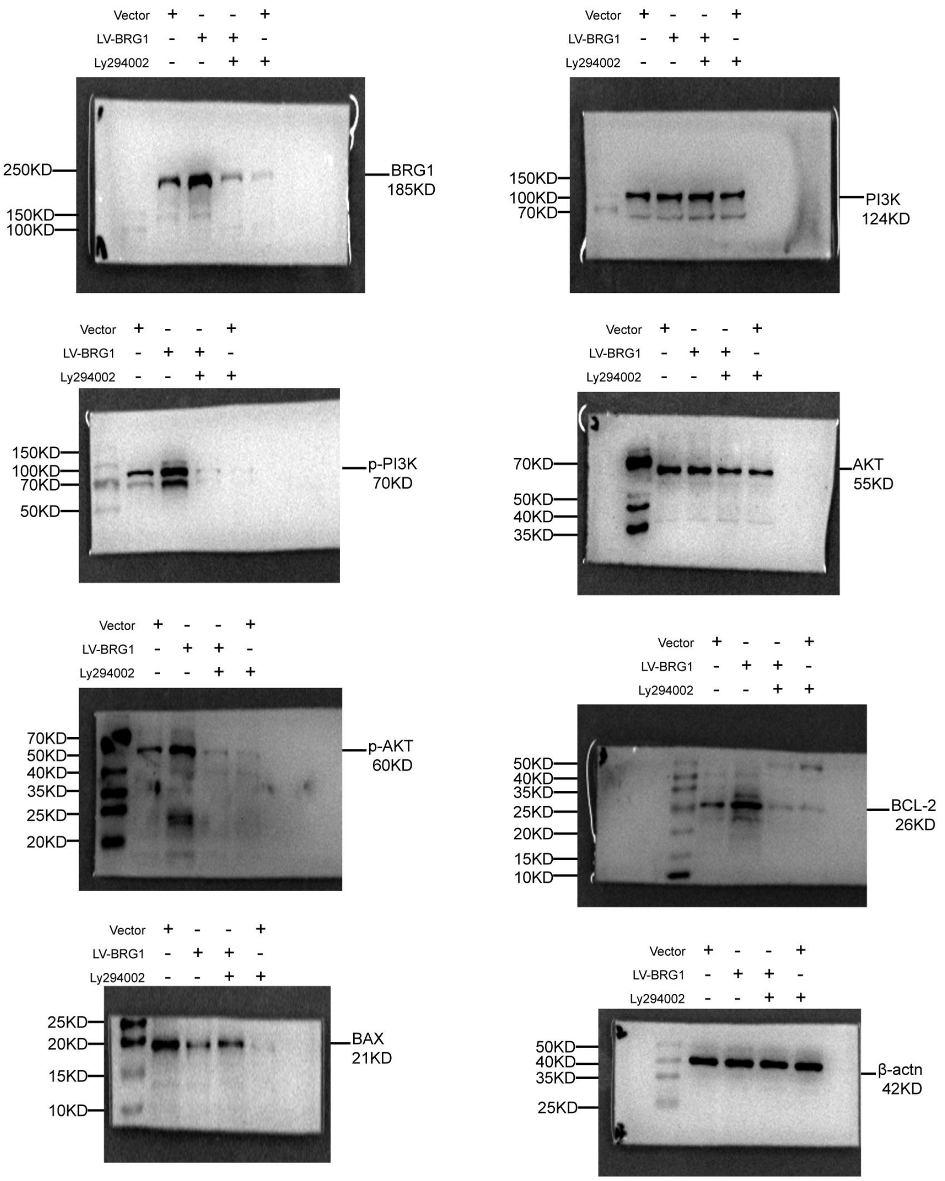

Supplement: Supplementary file 7 — Original western blots [file 41419_2024_6996_MOESM7_ESM.pdf]
